# Screening and Biological Evaluation of Soluble Epoxide Hydrolase Inhibitors: Assessing the Role of Hydrophobicity in the Pharmacophore-Guided Search of Novel Hits

**DOI:** 10.1021/acs.jcim.3c00301

**Published:** 2023-05-04

**Authors:** Javier Vázquez, Tiziana Ginex, Albert Herrero, Christophe Morisseau, Bruce D. Hammock, F. Javier Luque

**Affiliations:** † Departament de Nutrició, Ciències de l′Alimentació i Gastronomia, Facultat de Farmàcia i Ciències de l′Alimentació, Institut de Biomedicina (IBUB), Prat de la Riba 171, 08921 Santa Coloma de Gramenet, Spain; ‡ Pharmacelera, Parc Científic de Barcelona (PCB), Baldiri Reixac 4-8, 08028 Barcelona, Spain; § Department of Entomology and Nematology, and Comprehensive Cancer Center, University of California, Davis, One Shields Avenue, Davis, California 95616, United States; ∥ Departament de Nutrició, Ciències de l′Alimentació i Gastronomia, Facultat de Farmàcia i Ciències de l′Alimentació, Institut de Biomecidina (IBUB) and Institut de Química Teòrica i Computacional (IQTCUB), Prat de la Riba 171, 08921 Santa Coloma de Gramenet, Spain

## Abstract

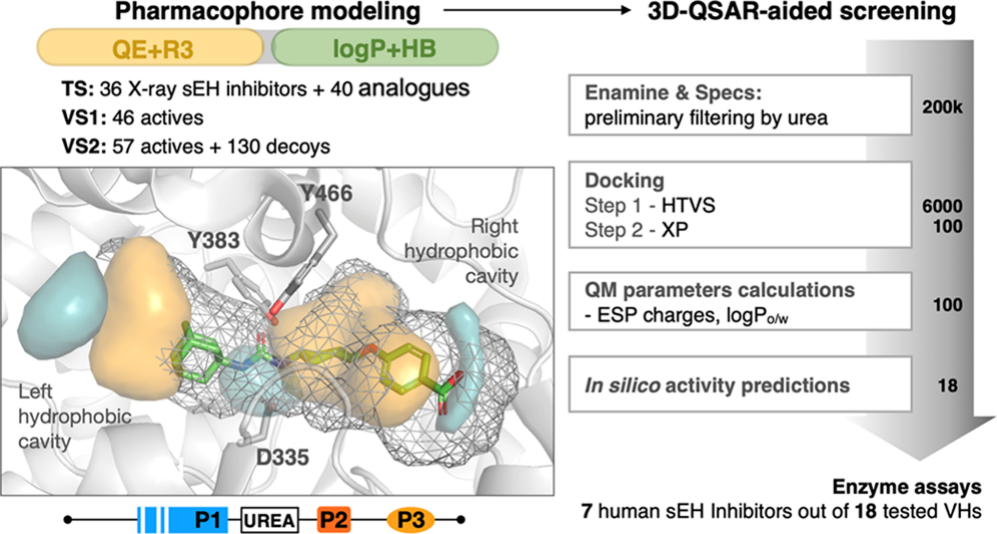

The human soluble epoxide hydrolase (sEH) is a bifunctional enzyme that modulates the levels of regulatory epoxy lipids. The hydrolase activity is carried out by a catalytic triad located at the center of a wide L-shaped binding site, which contains two hydrophobic subpockets at both sides. On the basis of these structural features, it can be assumed that desolvation is a major factor in determining the maximal achievable affinity that can be attained for this pocket. Accordingly, hydrophobic descriptors may be better suited to the search of novel hits targeting this enzyme. This study examines the suitability of quantum mechanically derived hydrophobic descriptors in the discovery of novel sEH inhibitors. To this end, three-dimensional quantitative structure–activity relationship (3D-QSAR) pharmacophores were generated by combining electrostatic and steric or alternatively hydrophobic and hydrogen-bond parameters in conjunction with a tailored list of 76 known sEH inhibitors. The pharmacophore models were then validated by using two external sets chosen (i) to rank the potency of four distinct series of compounds and (ii) to discriminate actives from decoys, using in both cases datasets taken from the literature. Finally, a prospective study was performed including a virtual screening of two chemical libraries to identify new potential hits, which were subsequently experimentally tested for their inhibitory activity on human, rat, and mouse sEH. The use of hydrophobic-based descriptors led to the identification of six compounds as inhibitors of the human enzyme with IC_50_ < 20 nM, including two with IC_50_ values of 0.4 and 0.7 nM. The results support the use of hydrophobic descriptors as a valuable tool in the search of novel scaffolds that encode a proper hydrophilic/hydrophobic distribution complementary to the target’s binding site.

## Introduction

Solvation is a crucial factor in determining the recognition and binding affinity between small ligands and their target receptors.^1^ The chemical structure of a drug-like candidate must encode an appropriate balance between hydrophilic and hydrophobic groups in order to sustain an adequate pharmacokinetic profile and to modulate the energy penalty due to desolvation upon ligand binding. Furthermore, the three-dimensional (3D) distribution of polar/apolar groups in the bioactive species must be optimal to guarantee the hydrophobic complementarity with the residues that shape the binding pocket. In this context, the effort spent in disclosing the relevant physicochemical features that determine the binding site druggability is not surprising.^2−10^ While no single physicochemical parameter consistently dominates the signature of binding pockets, these studies have revealed that the druggability is substantially modulated by descriptors of pocket shape, such as volume, depth and enclosure, and hydrophobicity, whereas polar or charged residues may be more relevant by imparting specificity, or by conferring structural stability to the ligand-bound complex through kinetic trapping. The knowledge gained from these studies has significant implications in key aspects of structure-based drug discovery, such as target assessment and validation^11−15^ and predicting the maximal affinity that a drug-like molecule may attain for a given binding site.^16−19^

The impact of hydrophobicity in determining the binding mode and affinity of ligands was early recognized by the development of hydrophobic-related descriptors for the analysis of structure–activity relationships (SAR). A cornerstone contribution was the pioneering work by Goodford leading to the GRID program,^20^ which computes the energy potential between a target molecule and suitable probes chosen to reflect distinct interactions, such as hydrogen bonding, shape, or hydrophobic contacts, laying the foundations for molecular interactions field (MIF) techniques.^21^ The explicit treatment of hydrophobic parameters has been included in a variety of computer-aided drug design tools, which can be exemplified by mentioning here (i) the molecular lipophilicity potential (MLP), which determines the influence of the constitutive fragments of a molecule on the surrounding environment using lipophilic contributions derived from experimental data,^22,23^ (ii) the comparative molecular similarity index analysis (CoMSIA), which was formulated to define 3D pharmacophores by considering originally electrostatic, steric, and hydrophobic fields and later supplemented with hydrogen-bond properties,^24,25^ (iii) FLAP, which is a GRID-related technique that identifies the discrete points associated with the most favorable interactions and exploits quadruplets of these points to define pharmacophoric fingerprints,^26^ and (iv) SiteMap, which was developed to characterize binding sites in terms of hydrophobic, hydrogen-bond donor, and hydrogen-bond acceptor maps.^27^ One of the most elaborate implementations of hydrophobicity for computer-aided drug design is the Hydropathic INTeractions (HINT) formalism,^28,29^ which was conceived as a non-Newtonian force field that describes polar and nonpolar interactions relying on hydrophobic atomic constants derived from experimental values of the *n*-octanol/water partition coefficient (log *P*_o/w_), assuming that the atomic contributions extracted from the solvation in these solvents reflect the polar and nonpolar interactions that mediate biomolecular recognition.^30,31^

The refinement of quantum mechanical (QM) continuum solvation methods^32^ and the accuracy achieved in predicting the solvation free energy^33,34^ has led to alternative approaches to characterize the 3D solvation pattern of small bioorganic compounds. As an example, the σ-profiles that describe the solvent reaction field generated by the solute’s charge distribution in the COSMO model have been used as an alternative descriptor to MIFs in order to measure the similarity between molecules and to explore the relationships with biological activity.^35,36^ On the other hand, we have developed a rigorous perturbative scheme^37^ to partition the solvation free energy into fragment contributions within the framework of the Miertus–Scrocco–Tomasi (MST) version^38,39^ of the integral equation formalism-polarizable continuum model (IEF-PCM) method.^40^ According to this scheme, the atomic contributions to the solvation in water and *n*-octanol are combined to derive atomic contributions to the log *P*_o/w._^41^ The QM-derived atomic hydrophobicities, denoted HyPhar, supplemented with descriptors of hydrogen-bond donor and acceptor features, have been used to generate hydrophobic fields in the three-dimensional quantitative structure–activity relationship (3D-QSAR)^42^ and screening of chemical libraries.^43,44^

While the results of these studies support the reliability of HyPhar parameters for providing a useful signature in SAR studies and to search for novel scaffolds, one may ask whether hydrophobic descriptors can be better suited than the standard (i.e., electrostatic charges and steric parameters) ones in computer-aided drug design, especially when the druggability of a binding site may be largely driven by the hydrophobic effect. Ultimately, a critical analysis of the topological and physicochemical features present in the target binding pocket might a priori be used to calibrate the suitability of distinct descriptors, since the nature and distribution of residues in the binding site should be informative about the expected contribution of distinct molecular properties (fields) to discriminate between actives and inactives or to disclose guidelines for lead optimization. Here, we address this question through a detailed analysis of the results obtained from the pharmacophore-guided screening of chemical libraries considering either the hydrophobic (HyPhar) descriptors or the electrostatic/steric fields for the human soluble epoxide hydrolase (hsEH). Choice of this enzyme obeys to several reasons, including the knowledge gained from medicinal chemistry studies covering a structurally diverse set of hsEH inhibitors, the availability of X-ray structures for ligand-hsEH complexes, and the peculiar features of the binding site (see below), in addition to the relevant pharmacological role played by the hsEH. In particular, beneficial biological effects on hypertension, atherosclerosis, diabetes, inflammation, and neuropathic pain have been linked to the inhibition of hsEH, even supporting the treatment for coronavirus-induced tissue damage.^45−49^

**Figure 1:**
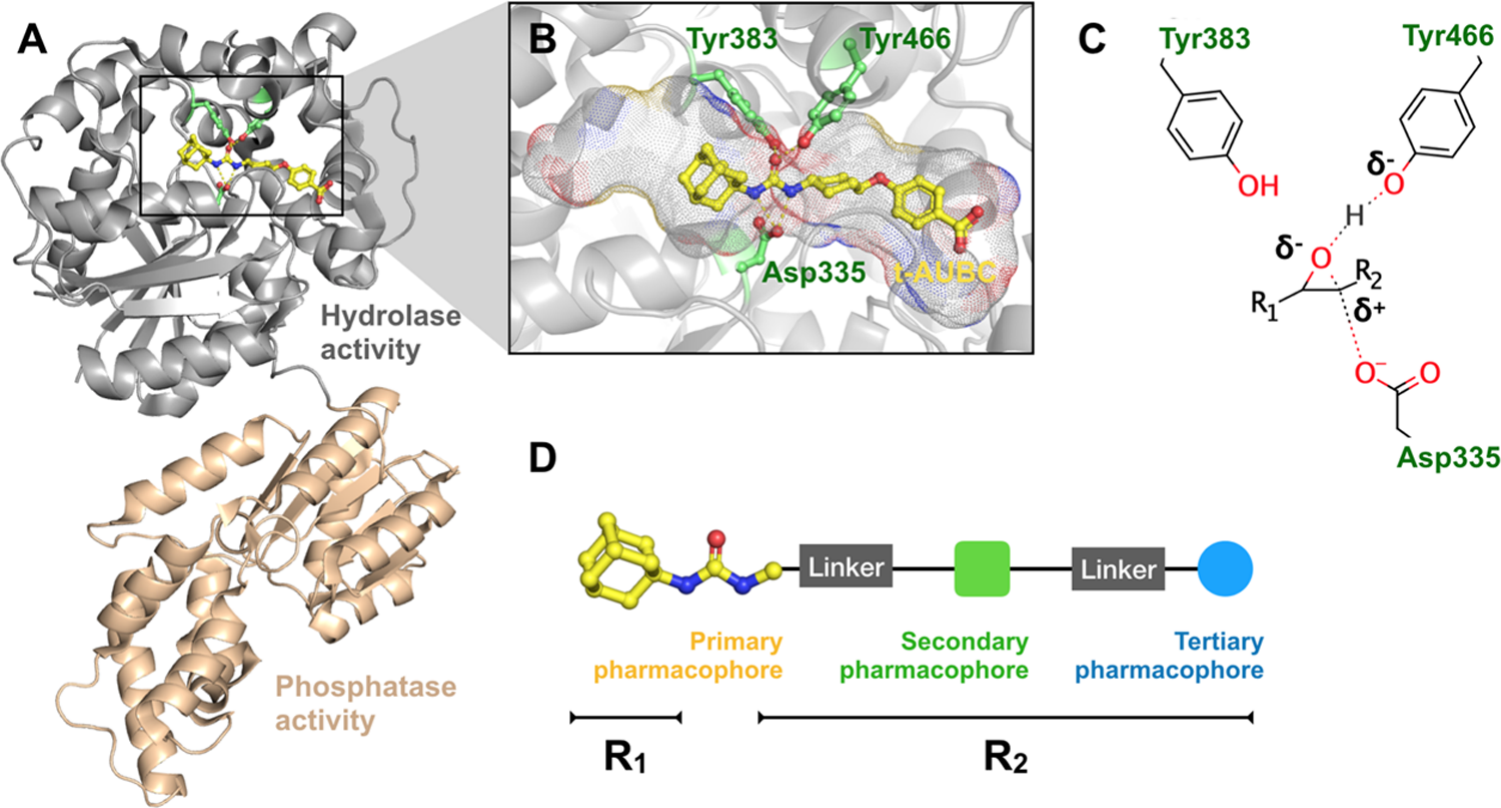
(A) Crystal structure of hsEH bound to 4-[(*trans*-4-{[(3s,5s,7s)-tricyclo[3.3.1.1∼3,7∼]dec-1-ylcarbamoyl]amino}cyclohexyl)oxy]benzoic acid (*t*-AUCB) (PDB ID: 3WKE). (B) Representation of the hydrophobic cavities that are located at the two sides of the catalytic triad. (C) Schematic arrangement of key residues (Tyr383, Tyr466, and Asp335) involved in the binding of the urea/amide moiety present in the hsEH inhibitors. (D) Main pharmacophore proposed in ref (51).

The hsEH enzyme carries epoxide hydrolase (EC 3.3.2.10) and phosphatase (EC 3.1.3.76) activities at the C- and N-termini, respectively (Figure 1A). The epoxide hydrolase function catalyzes the addition of water to epoxides leading to 1,2-diols, modulating the levels of regulatory epoxy lipids.^50^ For our purposes, it is worth noting that the binding cavity contains two wide hydrophobic sites, separated by a triad formed by Tyr383 and Tyr466, which activate the epoxide oxygen, and Asp335, which favors the epoxide ring opening (Figure 1B,C). Upon exclusion of this triad, 22 apolar residues shape the binding site, which only contains six polar residues, three being located at the edge of the pocket (Figure S1). Accordingly, a primary pharmacophore unit was early characterized by a 1,3-disubstituted urea/amide bound to a hydrophobic moiety such as an adamantan-1-yl group on one side, and completed on the other side with a secondary apolar or mildly polar moiety and a tertiary polar group to improve the metabolic stability and solubility (Figure 1D).^51^

Given the specific features of the hsEH binding pocket, this study reports a critical assessment on the suitability of different molecular descriptors for exploring compounds targeting the epoxide hydrolase pocket. To this end, 3D-QSAR models have been generated from a tailored list of crystallographic and noncrystallographic hsEH inhibitors considering either electrostatic and steric parameters or the QM-based HyPhar descriptors supplemented with hydrogen-bond parameters. Validation of the models has been accomplished by using external data reported in the literature as well as by evaluating their ability to discriminate between actives and decoys. Finally, a prospective study has been performed to identify potential hsEH inhibitors in chemical libraries and the predicted potencies compared with the inhibition activity measured in experimental assays. The aforementioned protocol allowed us to find several hsEH inhibitors in the low nM range, including two compounds with IC_50_ values of 0.4 and 0.7 nM.

## Methods

**Figure 2:**
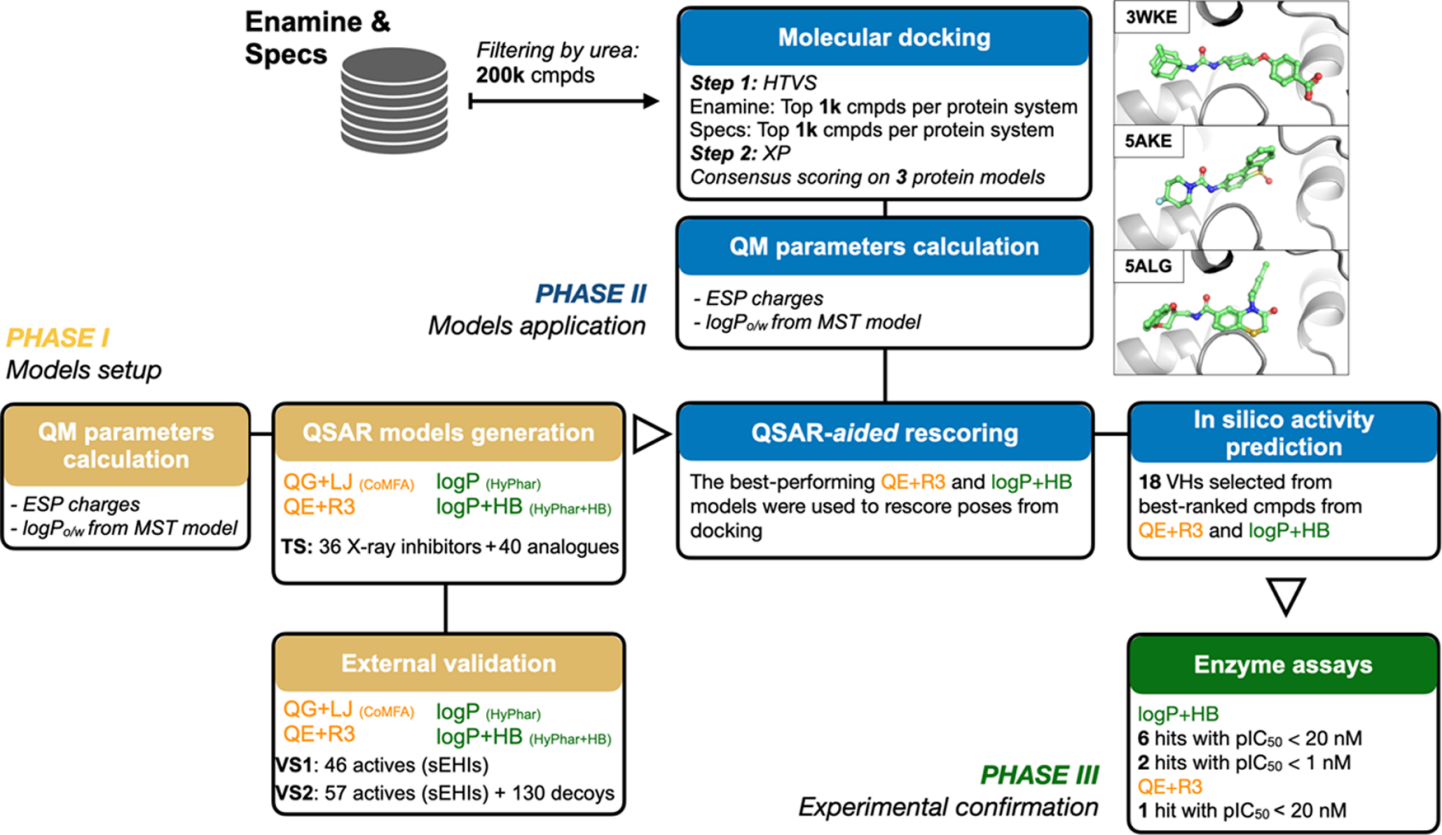
Detailed workflow of the *in silico–in vitro* protocol implemented in this study, which comprises three phases: (I) definition and validation of the pharmacophore models, (II) docking-based screening of chemical libraries and pharmacophore-guided reranking, and (III) experimental validation via enzymatic assays. QG + LJ denotes a pharmacophore built by combining Coulombic (Gasteiger charges; QG) and van der Waals (LJ) terms. QE + R3 stands for a pharmacophore obtained by combining QM-derived ESP (QE) atomic partial charges with the third power of the atomic radii (R3). Finally, log *P* corresponds to the model generated by using the HyPhar descriptors and log P + HB denotes the model obtained by combining HyPhar parameters with HB donor (HBD) and acceptor (HBA) descriptors.

The overall workflow adopted in this study is shown in Figure 2, which can be divided into three main phases: (I) definition and validation of pharmacophore models, (II) docking-based screening of chemical libraries and pharmacophore-guided reranking, and (III) experimental validation via enzymatic assays. In phase I, the first step comprises the generation of the pharmacophore models through the analysis of the X-ray crystallographic structures of small-molecule inhibitors available in the PDB. Next, the validation of the pharmacophore models was then performed using distinct external sets with a twofold purpose: (i) to check the suitability of the pharmacophores to rank the activity of small-molecule hsEH inhibitors reported in the literature, and (ii) to test their ability to discriminate between actives and decoys. Phase II comprises a prospective study to further assess the pharmacophore models; to this end, a docking-based screening of two chemical libraries was performed and the best-scored compounds were reranked using the pharmacophore models as a guide to select the best candidates. Finally, the last step of the protocol (phase III) was the analysis of the inhibitory potencies measured for a set of small-molecule inhibitors selected in a prospective study.

### Generation of 3D-QSAR Models

All of the available crystal structures deposited in the Protein Data Bank^52^ (PDB; last accession date: July 2021) corresponding to complexes of the hsEH bound to an inhibitor were downloaded and analyzed. Molecular fragments or ligands solved with low resolution were discarded to avoid biases in the generation of the 3D-QSAR models. Furthermore, a single copy of ligands present in more than one crystallographic structure was retained. This process led to a preliminary dataset of 36 protein–ligand X-ray structures (Table S1). In order to keep a proper distribution of inhibitory potencies in a range suitable for 3D-QSAR modeling, the dataset was further enriched with ligands (40 analogues) collected from the original papers that reported the X-ray structures used in this work (Table S1). Overall, 76 hsEH-inhibitor complexes, which covered a pIC_50_ range of about 6 log units, were finally compiled (p*K*_i_ values were used in few instances where the IC_50_ was not available).

The crystallographic ligands were extracted from the protein receptor and preprocessed using SYBYL X.^53^ For the noncrystallographic ligands, their 3D structures were manually built using the Tripos force field^54^ and energy-minimized by using the Powell optimization method with Gasteiger point charges, a nonbonded cutoff of 8.0 Å, and a dielectric constant of 2.0. A convergence criterion of 0.001 kcal mol^–1^ Å^–1^ or, alternatively, a maximum number of 1500 iterations was applied to calculations of the ligand’s geometry optimizations. The crystallographic ligands were centered in the hsEH catalytic binding site and aligned upon superposition of the protein backbone, paying attention to the proper alignment of the residues that shape the binding pocket around the catalytic triad. For each noncrystallographic ligand, the optimized geometry was aligned using the molecular skeleton of the parent crystallographic ligand as template.

The PharmQSAR software^42^ was used to generate four pharmacophores. Two models rely on the use of the HyPhar descriptors, which are based on the 3D map of log *P*_o/w_ atomic contributions computed by using the IEF-PCM/MST method parametrized at the B3LYP/6-31G(d) level.^39,55^ Calculations were performed using a locally modified version of Gaussian16.^56^ The pharmacophore models were obtained by exclusively treating the log *P*_o/w_ atomic contributions (log *P* model) or supplementing them with descriptors that denote the hydrogen-bond donor and acceptor nature of the polar atoms in the molecule (log *P* + HB model).^43^ The projection of the hydrophobic and HB fields onto the grid points was performed using an exponential decay function (eq 1; see ref (42) for details)
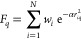
1where *F_q_* is the value of the projected field at grid point *q*, *i* stands for the summation index for *N* atoms of the molecule, *w*_*i*_ denotes the atomic hydrophobicity/HB component of atom *i*, α is the attenuation factor, which was set to 0.3, and *r*_iq_ is the distance between atom *i* of the molecule and the grid point *q*.

The other two models consist of the combination of electrostatic and steric descriptors, thus reflecting the fields adopted in other 3D-QSAR techniques.^24,57^ In one case, the atomic partial charges were determined at the B3LYP/6-31G(d) level by fitting the electrostatic potential computed from the electron density obtained upon solvation in *n*-octanol with the IEF-PCM/MST method, thus keeping a leverage between the level of theory used for deriving the HyPhar descriptors and the electrostatic potential-fitted (QE) atomic charges. On the other hand, the steric parameters were described using the atomic radii taken from the Tripos force field.^54^ Both electrostatic and steric fields were then determined from the projection of the partial charges and the third power of the atomic radius, respectively, using the exponential function in eq 1, leading to the QE + R3 model. For the sake of comparison, an additional model (QG + LJ) that combines Gasteiger charges and van der Waals parameters (taken from the Tripos force field) was also evaluated. Here, the molecular fields were generated by projecting the Coulombic and Lennard-Jones energies onto a regularly spaced grid around the aligned ligands following the CoMFA approach,^57^ in conjunction with a cutoff of 10 for the projected values.

The aligned inhibitors were placed in a 1.0 Å-spaced lattice with boundaries chosen to allow a minimum of 4 Å extension from the atoms in the molecules. The partial least-squares (PLS) method implemented in PharmQSAR was used to generate the 3D-QSAR models. The best model was selected according to the highest *q*^2^ obtained by using the leave-one-out (LOO) cross-validation method, and the lowest standard deviation error in prediction of the actual experimental values corrected by the number of degrees of freedom of the model (Spress).

### External Validation

Several validation sets were compiled to test the predictive capability of the 3D-QSAR models. First, four distinct series of hsEH inhibitors recently published in the literature were chosen to check the suitability of the pharmacophore models to predict the activity of 46 compounds containing distinct chemical scaffolds attached to the central urea/amide moiety (Table S2).^58−61^ The first series consists of 11 adamantyl urea derivatives where the adamantane group was replaced by alternative polycyclic hydrocarbons of different sizes.^58^ The second includes 18 oxaadamantyl ureas generated upon replacement of a methylene unit of the adamantane group by an oxygen atom to modulate solubility, permeability, and metabolic stability.^59^ The third encompasses nine urea-containing pyrazoles designed as COX-2/sEH dual inhibitors.^60^ Finally, eight amide-based compounds designed to simultaneously target hsEH and PPARγ were also included.^61^ Thus, a validation set of 46 inhibitors (named VS1), encompassing a pIC_50_ range from 5.8 to 9.4, was finally assembled.

The second validation set (VS2) was chosen to test the ability of the pharmacophore models to discriminate between actives and decoys. To this end, a set of 57 active compounds (pIC_50_ values comprised between 7.0 and 9.4) and 130 decoys was retrieved from the original data compiled in a previous study (Table S3),^62^ after exclusion of compounds lacking urea or amide units, which are relevant for the attachment to the catalytic triad in the enzyme (see above). Compounds unable to correctly accommodate the urea/amide moiety in the catalytic triad or characterized by internal atomic clashes in the docking pose were excluded. This led to a final set of 187 compounds among actives and decoys.

Since the lack of direct crystallographic information about the binding mode of these compounds may affect the predictive capability of the pharmacophore models, docking calculations were performed considering three hsEH protein models to account for the conformational flexibility of certain residues that shape the binding site on the ligand pose. A detailed comparison of the available X-ray structures led to the selection of PDB entries 3WKE,^63^5AKE, and 5ALG,^64^ which correspond to hsEH complexes with 4-[(*trans*-4-{[(3s,5s,7s)-tricyclo[3.3.1.1∼3,7∼]dec-1-ylcarbamoyl]amino}cyclohexyl)oxy]benzoic acid (*t*-AUCB), *N*-(5,5-dioxodibenzothiophen-2-yl)-4,4-difluoro-piperidine-1-carboxamide, and 4-[(3-chlorophenyl)methyl]-*N*-[[(3*S*)-2,3-dihydro-1,4-benzodioxin-3-yl]methyl]-3-oxidanylidene-1,4-benzothiazine-6-carboxamide.

The Glide docking program^65−67^ (Schrödinger Release 2021-3: Glide, Schrödinger, LLC, New York, NY, 2021) was used to generate the aligned 3D geometries for all of the molecules included in the two validation sets. The protonation state of the compounds was adjusted at pH 7.0. The extra precision (XP) scoring function was used in conjunction with a constraint introduced to assist the proper placement of the urea/amide moiety of the ligands in the hsEH catalytic cavity. The first 10 ranked poses were visually inspected to check for the presence of poses characterized by internal atomic clashes, which were excluded. For a given ligand, the final pose used for the 3D-QSAR modeling was selected as the best-ranked solution obtained among the docked poses in the three protein targets.

### Prospective Analysis

A final check of the log *P* + HB and QE + R3 pharmacophore models consisted of a prospective analysis to identify putative inhibitors of the hsEH. To this end, the Enamine (https://www.enamine.net) and Specs (https://www.specs.net) compound libraries were downloaded and the two libraries were filtered by using an *in-house* python script to retain compounds bearing the urea moiety, which is a key structural feature for the binding to the enzyme (Figure 1), leading to a dataset of 200,000 urea-containing compounds. The aligned 3D geometries for these compounds were generated by using a two-step docking procedure with Glide. First, a rapid screening of the compounds was performed by using the three hsEH targets mentioned above (PDB ID 3WKE, 5AKE and 5ALG) in conjunction with the high-throughput virtual screening (HTVS) protocol. The 1000 top ranked docking poses obtained for each hsEH target and each database (Enamine and Specs) were subjected to a second screening (6000 compounds) with the XP docking protocol. Following the procedure mentioned above for the external validation sets, the best-ranked solution obtained from the docking against the three protein targets was selected for each ligand, although they were visually inspected to eliminate poses with internal steric clashes. At the end, the 100 best-scored ligands were reranked using the pharmacophore models. The best nine compounds obtained separately from the log *P* + HB and QE + R3 pharmacophores were purchased and the inhibitory potency determined through in vitro assays.

### Experimental Determination of IC_50_

The IC_50_ values of the 18 compounds selected from the log *P* + HB and QE + R3 pharmacophore ranking were determined using a fluorescence-based assay^68^ with either purified recombinant hsEH, mouse sEH (msEH), or rat sEH (rsEH). The enzyme was incubated at 30 °C with the inhibitors ([I]_final_ = 0.4–50,000 nM) for 5 min in 100 mM sodium phosphate buffer (200 μL, pH 7.4) containing 0.1 mg/mL of ovine serum albumin (BSA) and 1% dimethyl sulfoxide (DMSO). The substrate (cyano(6-methoxynaphthalen-2-yl)methyl((3-phenyloxiran-2-yl)methyl)carbonate; CMNPC) was then added ([S]_final_ = 5 μM). The activity was assessed by measuring the appearance of the fluorescent 6-methoxynaphthaldehyde product (λ_ex_ = 330 nm, λ_em_ = 465 nm) every 30 s for 10 min at 30 °C on a SpectraMax M2 (Molecular Devices, CA). The results were obtained by regression analysis from a linear region of the curve.

## Results and Discussion

In the following paragraphs, the results obtained in the generation of the pharmacophore models, their validation with external datasets, and the results of the prospective study will be discussed.

### Generation of Pharmacophore Models

The training set utilized for the generation of the pharmacophore model consisted of 76 hsEH-inhibitor complexes spanning inhibitory potencies ranging from 3.3 to 9.6 pIC_50_ units (see Figure S3). Almost half of them were taken directly from X-ray structures deposited in the PDB (Table S1). In this case, the inhibitors were aligned upon superposition of the protein backbone, and in few cases, slight adjustments were made after visual inspection to maximize the overlay of key residues, particularly regarding the interaction of the urea/amide moiety of the inhibitor with the triad formed by Asp335, Tyr383, and Tyr466, and the spatial arrangement of specific moieties with poorly defined electrons in the X-ray electron density (compounds mol22, mol28, and mol29). To avoid the presence of gaps in the distribution of inhibitory potencies, noncrystallographic, but structurally congeneric ligands were also added (Table S1). In this case, the energy-minimized geometry was manually superposed using the molecular skeleton of the parent crystallographic ligand as the template. As a final remark, it must be noted that although the inhibition activity compiled in the Supporting Information for the final dataset is indicated as the pIC_50_ data, the inhibitory potency reported in the literature for compounds TR_mol12, TR_mol13, and TR_mol44 was determined for the racemate, and the data listed for compounds TR_mol10, TR_mol11, and TR_mol65–68 correspond to the inhibition constant (p*K*_i_). While this may introduce some noise in the experimental potencies, it is not expected to have a significant impact on the pharmacophore models due to the size of the final training set and the range of inhibitory potencies spanned by the compounds.

**Figure 3:**
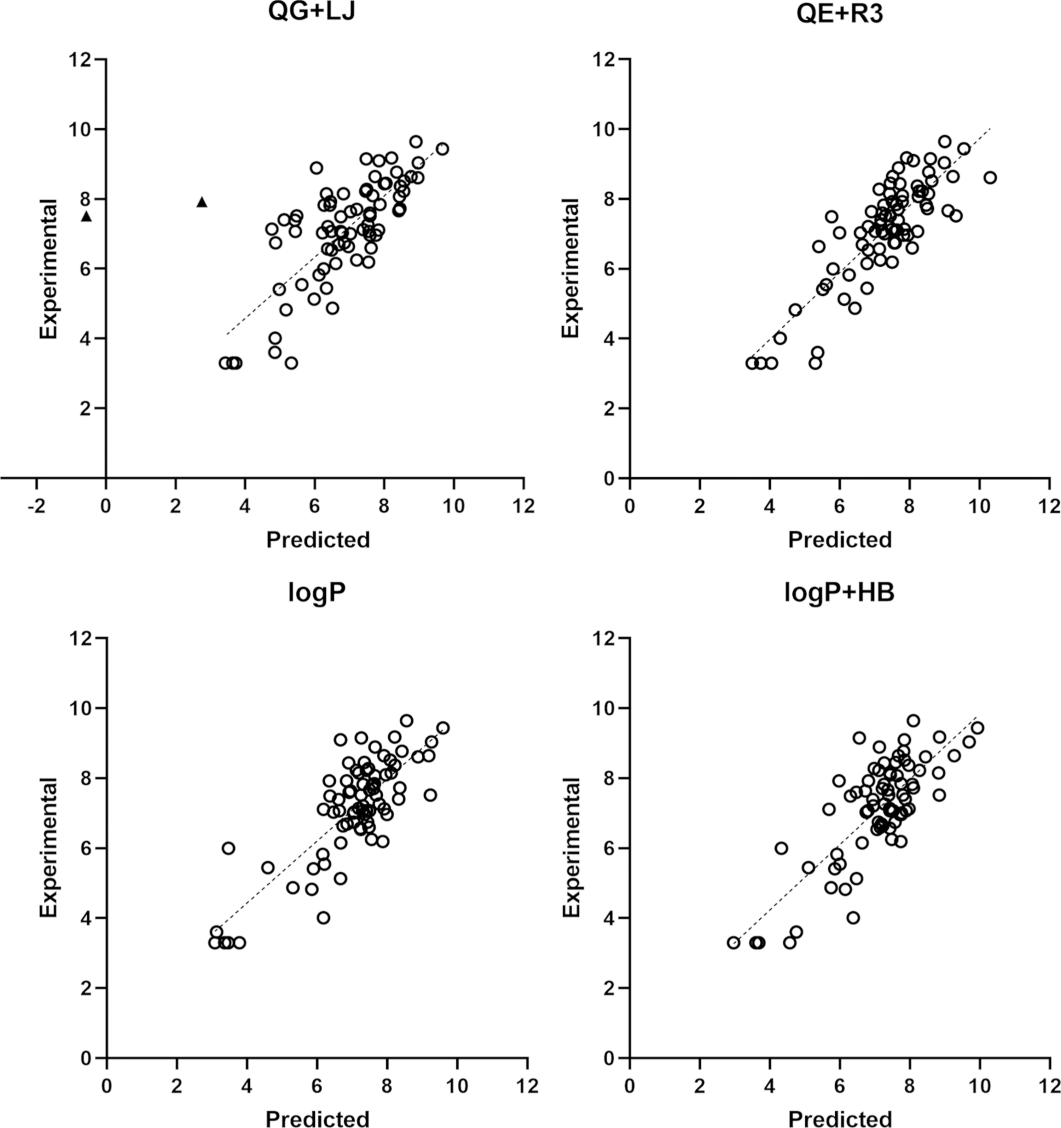
Comparison of the experimental pIC_50_ values versus the predicted (LOO cross-validated) ones using the four pharmacophore models. The two compounds that exhibit large deviations from the regression model in the QG + LJ pharmacophore are shown as black triangles. The ideal regression line is shown as a dashed line.

The pharmacophore models were built using the standard PLS algorithm and subjected to a leave-one-out (LOO) cross-validation to identify the optimal number of latent variables so that the best model was chosen according to the minimum in the sum of the squared differences between predicted and actual pIC_50_ values. The statistical parameters obtained for the four pharmacophores are shown in Table 1, and the comparison between experimental and cross-validated pIC_50_ data for the training set is shown in Figure 3 (see also Figure S2 for the comparison between experimental and fitted pIC_50_ values).

**Table 1:** Summary of Statistical Parameters of the Pharmacophore Models Obtained for the Compounds in the Training Set^a^

parameter	QG + LJ	QE + R3	log *P*	log *P* + HB
*N*_c_	7	7	3	3
field (%)	QG: 27	QE: 54	log *P*: 100	log *P*: 83
	LJ: 73	R3: 46		HBD: 13
				HBA: 4
Regression Model *y* = *mx* + *n*				
*m*	0.54 (0.88)^b^	0.93	0.88	0.93
*n*	3.51 (1.07)^b^	0.33	0.89	0.49
*q*^2^	0.34 (0.59)^b^	0.68	0.66	0.62
*Spress*	1.27	0.88	0.89	0.94
Regression Model *y* = *cx*				
*c*	1.03	0.98	1.01	1.00
*r*^2^	0.96	0.99	0.99	0.98

a *N*_c_: optimum number of components selected on the basis of the leave-one-out cross-validation method; *q*^2^: leave-one-out cross-validation correlation coefficient; *Spress*: standard deviation error in prediction of the actual experimental values corrected by the number of degrees of freedom of the model; *r*^2^: Pearson’s correlation coefficient between experimental and predicted values.

b *N*_c_: values obtained upon exclusion of compounds TR_mol19 and TR_mol69.

The cross-validated pIC_50_ values exhibit a nice correlation with the experimental data. The scaling coefficient of the regression equations through the origin (*y* = *cx*) ranges between 1.00 and 1.03, and the correlation coefficient (*r*^2^) is larger than 0.96. Nevertheless, one can notice that the regression equation through the origin obtained for the QG + LJ model is not close to the optimal regression (*y* = *mx* + *n*), which has a cross-validated regression (*q*^2^) of 0.34. This reflects the large deviation observed in the predicted values for two compounds (compounds TR_mol19 and TR_mol69; Figure 3) whose exclusion increases the value of *q*^2^ to 0.59. In contrast, the use of the ESP charges in the QE + R3 model improves the overall performance, leading to a closer resemblance between the optimum regression equation (*q*^2^ = 0.68) and the regression line through the origin. On the other hand, the HyPhar descriptors lead to models of statistical quality comparable to the QE + R3 model, as the correlation coefficients of the cross-validated model (*q*^2^) are 0.69 and 0.62 for models log *P* and log *P* + HB, respectively. It is worth noting, nevertheless, that the number of components is reduced from 7 in the QE + R3 model to only 3 in both log *P* and log *P* + HB ones, which supports the ability of the latter descriptors to identify the molecular features that determine the inhibitory potency. Furthermore, the relevance of the hydrophobicity can be noted in the weight of the log *P* field in the log *P* + HB model, which amounts to 83%, whereas the contribution of the HB donor and acceptor fields is 13 and 4%, respectively. As a last remark, we also examined the effect of excluding data corresponding to racemic compounds (TR_mol12, TR_mol13, and TR_mol44) and those characterized by the inhibition constant (p*K*_i_) instead of pIC_50_. The statistical parameters of the pharmacophore models derived for these subsets (Tables S4 and S5) are little affected and retain the trends discussed above for the whole set of compounds.

**Figure 4:**
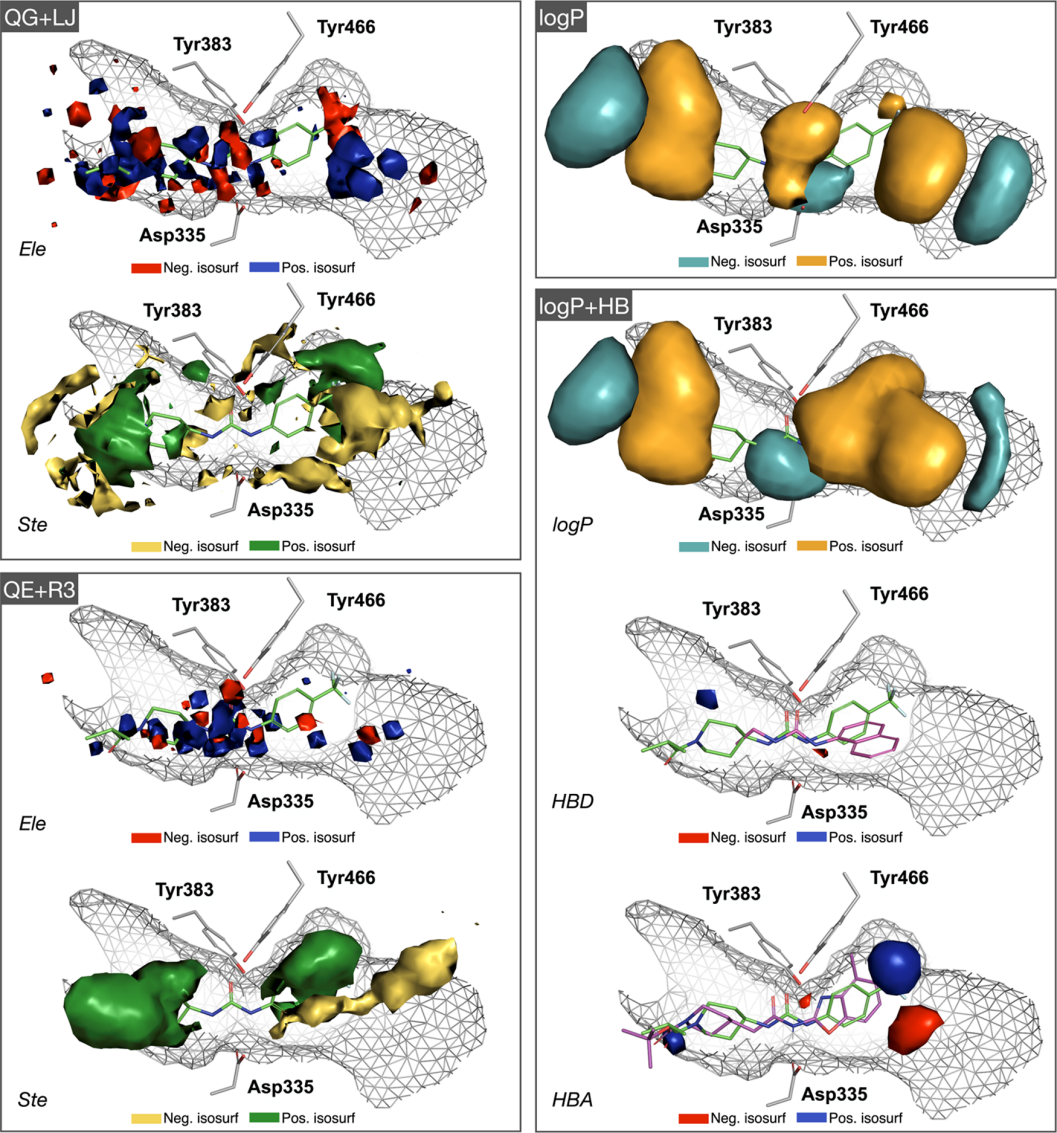
Isocontour maps for the four pharmacophoric models QG + LJ (QG: +5/–3; LJ: +150/–50), QE + R3 (QE: +0.1/–0.2; R3: +2/–1), log *P* (log *P*: +5/–3), and log *P* + HB (log *P*: +3/–3; HBD: +0.5/–3.3; HBA: +5/–1.6). The shape of the binding site (PDB ID: 3WKE) is represented as a gray wireframe. For an easier interpretation of the isocontour maps, residues forming the catalytic triad (Tyr383, Tyr466, and Asp335) are also shown as gray sticks. The most potent inhibitor from the X-ray subset of the training set (TR_mol10; in green sticks pIC_50_ = 9.2; see Table S1) is shown as green sticks. For the sake of comparison, two low-potency inhibitors, TR_mol7 (in magenta sticks; pIC_50_ = 4.0) and TR_mol47 (in magenta sticks; pIC_50_ = 6.6) are also shown in the HBD and HBA maps.

Since a 3D-QSAR model should provide an easily interpretable graphical representation of physicochemical properties relevant for the biological activity, a graphical comparison of isocontour maps derived from the pharmacophore models is valuable to gain insight into the predictive potential of the IEF-PCM/MST-derived hydrophobic descriptors (Figure 4). Regarding the QG + LJ and QE + E3 models, the MIFs generated from the steric descriptors are highly similar, as they show two (green) areas, located on each of the cavity subsites that are separated by the catalytic triad, which would contribute favorably to the inhibitory activity, and a large (yellow) region that would disfavor the presence of chemical groups in the ligand. The pharmacophore generated from the atomic partial charges exhibits larger dissimilarities, although a common feature is the fragmented distribution pattern of electrostatically favorable/unfavorable contacts in the binding pocket, which makes it difficult to envisage precise guidelines for assisting the analysis of structure–activity relationships.

In contrast with the previous trends, a more consistent and readable profile is provided by the projection of the HyPhar descriptors. In particular, the pharmacophore that emerges from the projection of the log *P* field delineates regions where the presence of apolar (orange)/polar (cyan) groups in the ligand would contribute favorably to the inhibitory potency. Notably, the 3D distribution of regions encompassing positive/negative isocontours is highly similar in the pharmacophores derived from log *P* and log *P* + HB models, in agreement with the large weight of the log *P* field in this latter pharmacophore (83%; Table 3). This reflects the modest contribution played by the HBD (13%) and HBA (4%) fields to the final PLS model. With regard to the HB fields, the presence of small negative (red) regions can be noticed located below the ligand-anchoring Tyr residues and above Asp335, which can be interpreted in terms of the requirements for a proper arrangement of HB donor (NH) and acceptor (carbonylic oxygen) groups of the ligand for attachment to the catalytic triad, as exemplified for compounds TR_mol7 (pIC_50_ = 4.0) and TR_mol47 (pIC_50_ = 6.6) in Figure 4. Finally, let us note that the information encoded in the Hyphar-derived pharmacophores reflects the chemical features proposed for a prototypical inhibitor,^51^ which should consist of a central urea acting as the anchoring group to the catalytic triad as well as a linker for two bulky hydrophobic fragments (corresponding to positive areas of the log *P* field shown as bright orange isocontours), which in turn could be decorated with some polar groups (i.e., carboxyl-) at the two edges of the binding cavity (negative areas of the log *P* field shown as cyan isocontours).

### External Validation (VS1): Prediction of the pIC_50_ Data

Four distinct series of hsEH inhibitors published recently in the literature^58−61^ were chosen as an external set to check the suitability of the pharmacophore models to rank the potency of potential inhibitors of this enzyme (Table S2). The range of pIC_50_ values determined for the compounds reported in each series varies from 1.7 to 2.2. It is not reasonable to expect that a 3D-QSAR model can achieve this level of accuracy, which is even challenging for more advanced methods, such as free energy perturbation.^69−71^ Therefore, the four series, which comprise 46 compounds encompassing a range of inhibitory potencies (pIC_50_) between 5.8 and 9.4 (see Figure S3), were considered together in the analysis of the pharmacophore models (see Table 2). Indeed, this is close to the range of pIC_50_ values of the compounds included in the training set. Furthermore, since mixing of data from different studies may introduce a random noise due to the usage of different experimental protocols and techniques, choice of these studies was also motivated by the internal consistency in the experimental assays, which in all cases resorted to a fluorescence-based assay that detects the formation of 6-methoxynaphthaldehyde by hsEH, although differences regarding the use of the substrate (CMNPC: cyano(2-methoxynaphthalen-6-yl)methyl *trans*-(3-phenyl-oxyran-2-yl)methyl carbonate; PHOME: (3-phenyl-oxiranyl)-acetic-acid-cyano-(6-methoxy-naphthalen-2-yl)-methylester) and the incubation time (from 5 to 45 min) were also noticed. Finally, a third factor to be considered is the definition of the binding pose, which was determined from docking calculations but for the study reported by Lillich et al.,^61^ where compounds were manually superposed taking advantage of the X-ray crystallographic structure available for the complex between hsEH and compound VS1_mol42. The assumption of a common binding mode for each set of inhibitors is based on the fact that they involve structurally congeneric compounds, which exhibit chemical differences localized in a specific region of the molecular skeleton.

**Table 2:** Experimental and Calculated pIC_50_ Values for the 46 Compounds Included in the External Validation Set (VS1)

compound	experimental	QG + LJ	QE + R3	log *P*	log *P* + HB
VS1_mol1	6.0	7.5	9.3	6.4	6.5
VS1_mol2	6.3	7.6	8.0	6.4	6.3
VS1_mol3	6.3	7.8	8.1	7.3	6.8
VS1_mol4	6.8	7.8	8.5	7.3	7.1
VS1_mol5	7.3	7.9	8.4	7.5	7.1
VS1_mol6	6.7	7.9	8.0	6.8	6.8
VS1_mol7	6.7	7.9	8.0	6.7	6.5
VS1_mol8	6.5	7.9	8.0	6.5	6.4
VS1_mol9	7.1	7.7	8.3	7.6	7.4
VS1_mol10	6.6	7.5	7.2	6.2	6.31
VS1_mol11	8.0	5.6	8.4	7.9	7.6
VS1_mol12	8.2	3.0	6.0	7.8	8.2
VS1_mol13	7.1	2.0	6.0	7.0	7.1
VS1_mol14	7.1	2.2	5.9	7.1	7.5
VS1_mol15	7.6	4.2	5.7	8.6	8.5
VS1_mol16	8.4	6.2	7.3	7.7	7.4
VS1_mol17	8.5	3.1	6.2	7.9	7.4
VS1_mol18	8.0	3.7	3.6	8.7	8.3
VS1_mol19	9.3	3.0	6.5	8.6	8.8
VS1_mol20	9.3	3.8	7.1	9.3	9.3
VS1_mol21	9.4	6.6	6.2	8.0	7.5
VS1_mol22	9.4	6.6	6.4	8.4	7.9
VS1_mol23	8.5	6.0	6.1	7.8	7.8
VS1_mol24	8.5	6.5	6.3	7.5	7.8
VS1_mol25	8.1	7.3	7.6	8.5	8.2
VS1_mol26	8.1	6.8	7.9	8.3	7.9
VS1_mol27	9.3	7.9	8.5	8.6	8.0
VS1_mol28	8.2	7.8	8.2	7.6	7.5
VS1_mol29	7.7	7.5	7.3	7.3	7.3
VS1_mol30	8.5	8.7	9.4	8.2	7.8
VS1_mol31	8.1	8.5	9.6	8.1	7.8
VS1_mol32	7.7	6.8	6.8	7.4	7.9
VS1_mol33	6.1	6.2	6.4	6.4	6.4
VS1_mol34	6.0	6.1	6.3	6.3	6.4
VS1_mol35	5.8	6.6	6.6	6.1	6.3
VS1_mol36	7.3	7.1	7.0	7.7	6.9
VS1_mol37	7.7	–0.7	7.1	7.5	7.6
VS1_mol38	7.5	7.8	7.7	6.5	6.4
VS1_mol39	6.5	7.1	7.2	7.3	7.5
VS1_mol40	8.3	7.9	8.0	6.8	7.5
VS1_mol41	8.5	9.1	8.2	7.2	7.7
VS1_mol42	8.1	8.8	8.2	7.4	7.8
VS1_mol43	8.3	7.8	7.6	7.5	8.1
VS1_mol44	8.2	7.5	7.5	7.6	7.8
VS1_mol45	6.8	7.2	7.4	6.3	6.6
VS1_mol46	7.9	7.4	7.5	6.4	6.9
Regression Model *y* = *mx* + *n*					
*m*		–0.07	–0.09	0.96	1.11
*n*		8.1	8.3	0.5	–0.5
*r*^2^		0.02	0.01	0.59	0.62
					
Regression Model *y* = *cx*					
*c*				1.03	1.04
*r*^2^				0.59	0.62

**Figure 5:**
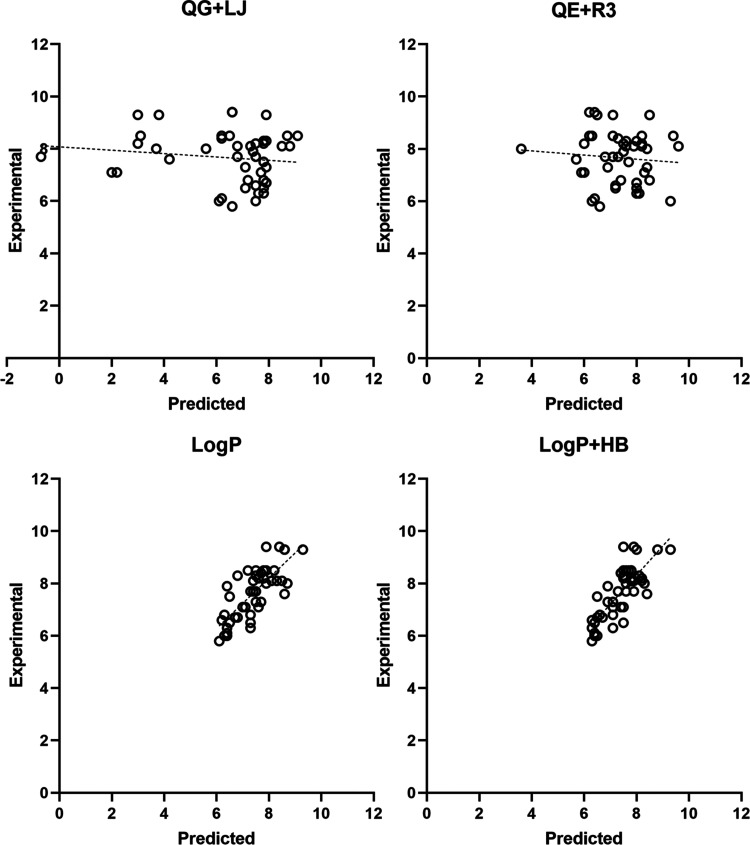
Comparison of the experimental pIC_50_ values versus the predicted ones determined using the four pharmacophore models for the compounds in the validation set (VS1). The ideal regression line is shown as a dashed line.

The comparison of experimental/predicted inhibitory potencies of the compounds is shown in Figure 5. In spite of the scattered distribution of the compounds in the models obtained with log *P* and log *P* + HB fields, one may notice a correlation between experimental and estimated values, which is absent in the graphical representations obtained for the QG + LJ and QE + R3 models. Indeed, it is worth noting that the two regression models obtained for both log *P* and log *P* + HB pharmacophores exhibit slopes that are close to unity (see Table 2), and that the *r*^2^ parameter of the regression model through the origin is close to the value obtained for the optimal regression, thus giving confidence to the robustness of these pharmacophores for ranking of putative hsEH inhibitors. At this point, let us note that, beyond a high value of cross-validated *r*^2^, an additional criterion to support a predictive model is the identification of a small angle between the lines generated by equations *y* = *mx* + *n* and *y* = *cx*, which implies closeness of the correlation coefficients from the two regression models and of the slope of the regression line through the origin to 1.^72^ Furthermore, it is also interesting to note the similar distributions obtained for log *P* and log *P* + HB models, which are consistent with the lower weight of the hydrogen-bond descriptors to the pharmacophore model (see above).

### External Validation (VS2): Discrimination between Actives and Decoys

To further explore the predictive ability of the pharmacophore models, we have examined the reliability to discriminate between actives and decoys taking advantage of a recent study that compiled a literature survey for known sEH inhibitors and confirmed inactive (decoys) compounds.^62^ Out of the published structures, 57 chemically diverse urea/amide-containing inhibitors with IC_50_ values from the subnanomolar range up to 13.400 nM as well as 130 confirmed inactive compounds (Table S3) were considered in this test.

**Figure 6:**
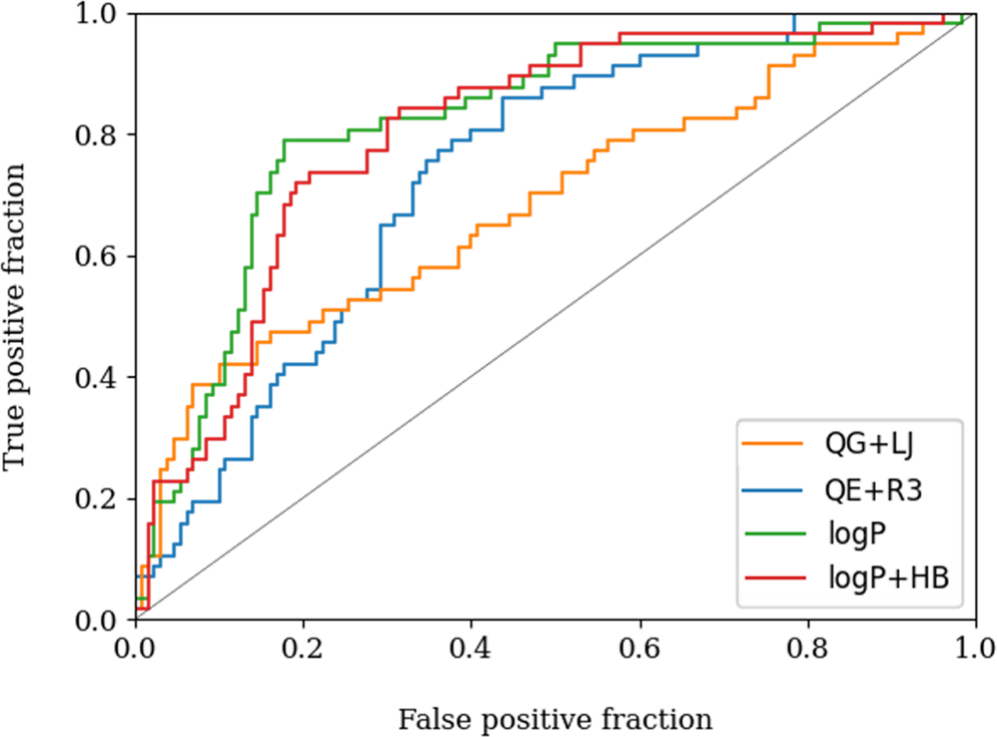
ROC curves obtained for the recovery of actives included in the external validation set (VS2). Actives and decoys are ordered based on their activity values.

The receiver operator characteristic (ROC) curve was used to measure the performance of the pharmacophore models to recover the actives from the whole dataset. As can be seen in Figure 6, the ROC curves obtained for the log *P* and log *P* + HB models consistently showed a better performance compared to the QG + LJ and QE + R3 ones. The area-under-the-curve (AUC) values for the two log *P*-based models are 0.81 and 0.80, respectively, whereas the values determined for QE + R3 and QG + LJ are 0.73 and 0.68. The comparison between early enrichments is more delicate due to the low number of decoys included in the set of compounds. However, for the compounds included in the first 5%, the ROC value obtained for the QG + LJ model is 5.3, which compares with the values obtained by using the log *P* and log *P* + HB pharmacophores (5.3 and 7.9, respectively). Nevertheless, the success of the QG + LJ model in discriminating actives/inactives is reduced drastically as the ranking progresses.

**Figure 7:**
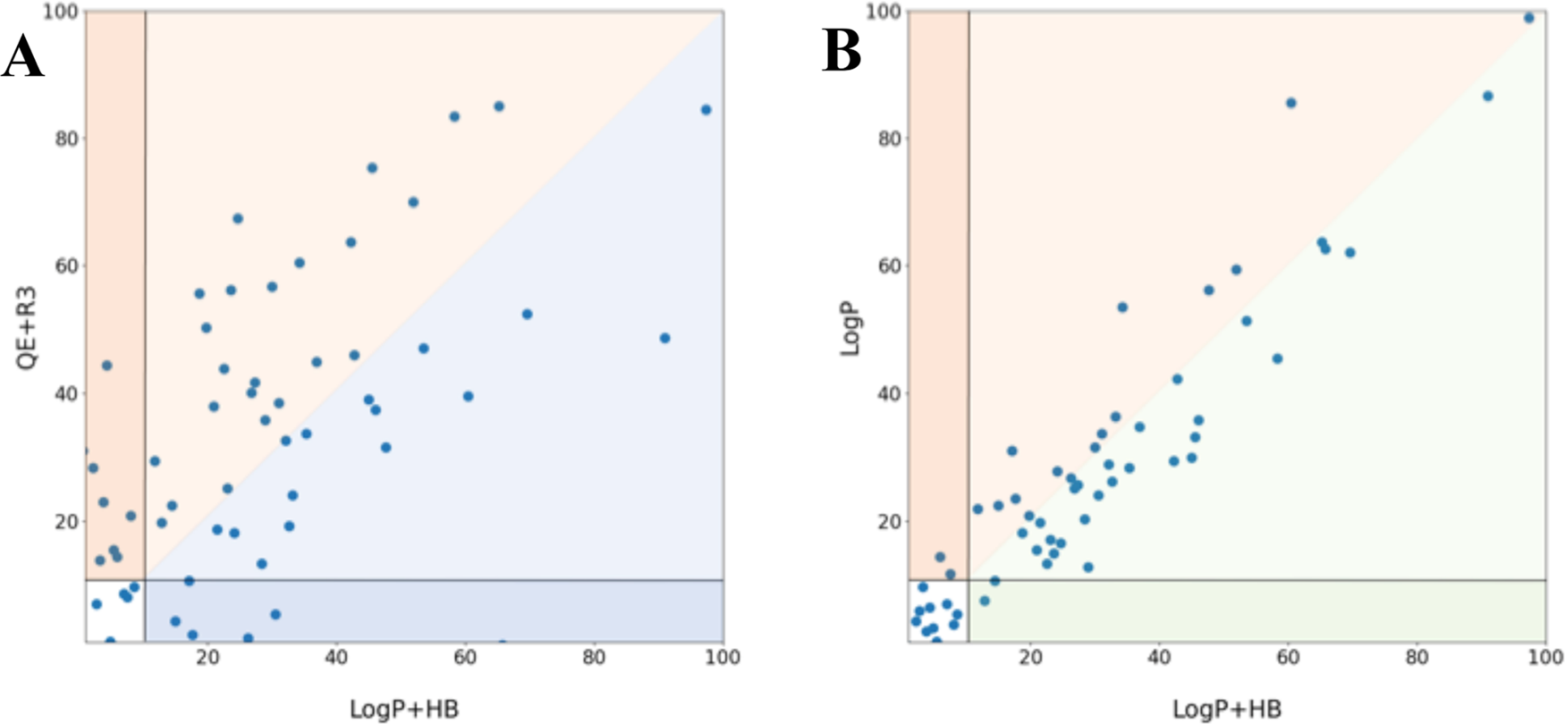
Distribution of the active compounds for the VS2 dataset as ranked by the log *P* + HB model versus the actives recovered by using the (A) QE + R3 and (B) log *P* models. The position of the actives recovered for these methods has been normalized relative to the total number of compounds in every set (from 1 to 100%). The areas where the actives are better ranked by QE + R3, log *P*, and log *P* + HB models are depicted in blue, orange, and green, respectively.

It is also worth noting that the active compounds recovered from the pharmacophore models built using either electrostatic + steric fields or log *P*-based ones exhibit notorious differences. This behavior can be stated in Figure 7, which compares the position (normalized from 1 to 100%) occupied by all active compounds recovered by QE + R3, log *P*, and log *P* + HB pharmacophores. Thus, five identical compounds are identified as actives within the first 10% by the QE + R3 and log *P* + HB models (Figure 7A). However, eight additional actives recovered with the log *P* + HB model in the first 10% are scattered up to 45% when the QE + R3 pharmacophore is considered. On the contrary, four additional actives found in the first 10% according to the QE + R3 descriptors appear as hits in the first 30% with the log *P* + HB model. This suggests that the QE + R3 and log *P* + HB pharmacophores tend to prioritize the selection of distinct chemical spaces, which may in turn be valuable to enrich the feasibility for disclosing novel scaffolds. On the other hand, a close correspondence is observed between the actives recovered by using the log *P* and log *P* + HB models, as expected from the larger weight of the 3D distribution of hydrophobicity in the latter pharmacophore model (Figure 7B).

### Prospective Analysis

As a final test about the performance of the electrostatic + steric and hydrophobic fields to explore the chemical space in the search of novel hits, a dataset of 200,000 urea-containing compounds were screened using a two-step docking protocol and three hsEH targets (PDB ID 3WKE, 5AKE, and 5ALG). This was motivated to take into account the distinct arrangements observed in the side chains of few residues, which are mainly located at the periphery of the binding pocket, upon comparison of the X-ray crystallographic structures available for ligand-hsEH complexes (see Methods). Let us note, however, that other approaches could have been adopted to account for the ligand-induced receptor flexibility, such as IFREDA, which is a computational merging-and-shrinking procedure implemented in the ICM docking program to account for sampling the conformational space of the receptor, even in cases of large loop movements.^73^

**Figure 8:**
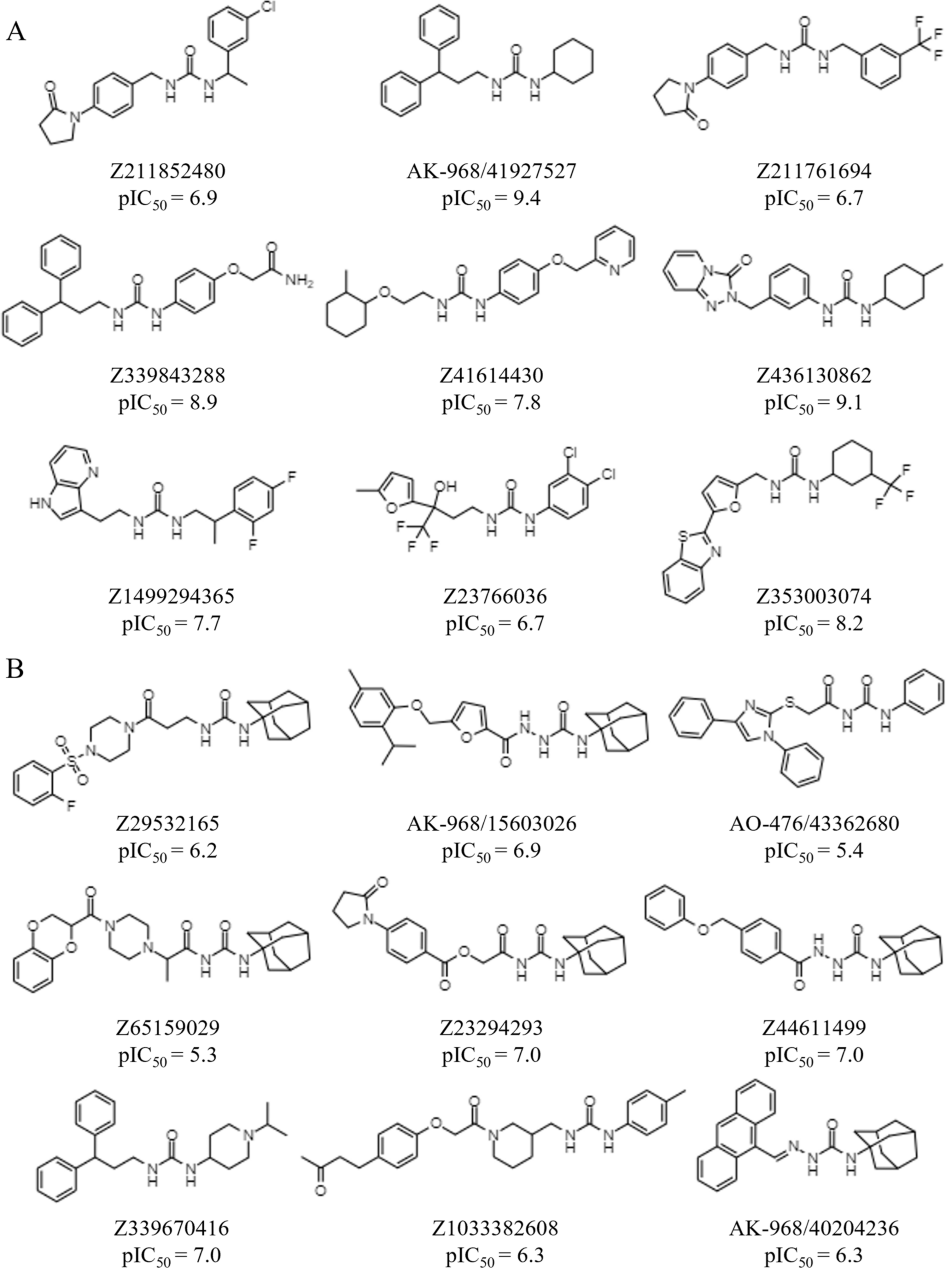
Chemical structures of the compounds selected using (A) log *P* + HB and (B) QE + R3 pharmacophores as potential candidates to inhibit the hsEH enzyme. The inhibitory potency measured in experimental assays against hsEH is given as pIC_50_ values.

At the end of the docking process, the poses of the top 100 ligands were reranked using the QE + R3 and log *P* + HB pharmacophores. The use of this hybrid strategy that combines docking and pharmacophore reranking aims to exploit the available information of both ligands and target to enhance the success of drug discovery projects.^74^ Finally, the inhibitory potency of the best nine compounds ranked with each pharmacophore was determined using a fluorescence-based in vitro assay.^68^ The two-dimensional (2D) structures for the virtual hits selected for experimental evaluation from QE + R3 and log *P* + HB pharmacophores are shown in Figure 8 (see Table S6 for information about their physicochemical properties). In spite of the apparent chemical resemblance afforded by the urea moiety, it is worth noting that the compounds selected from the two pharmacophores appear as distinct groups when categorized according to their structural similarity (Morgan fingerprint radius 2) in a principal component analysis (see Figure S4).

**Figure 9:**
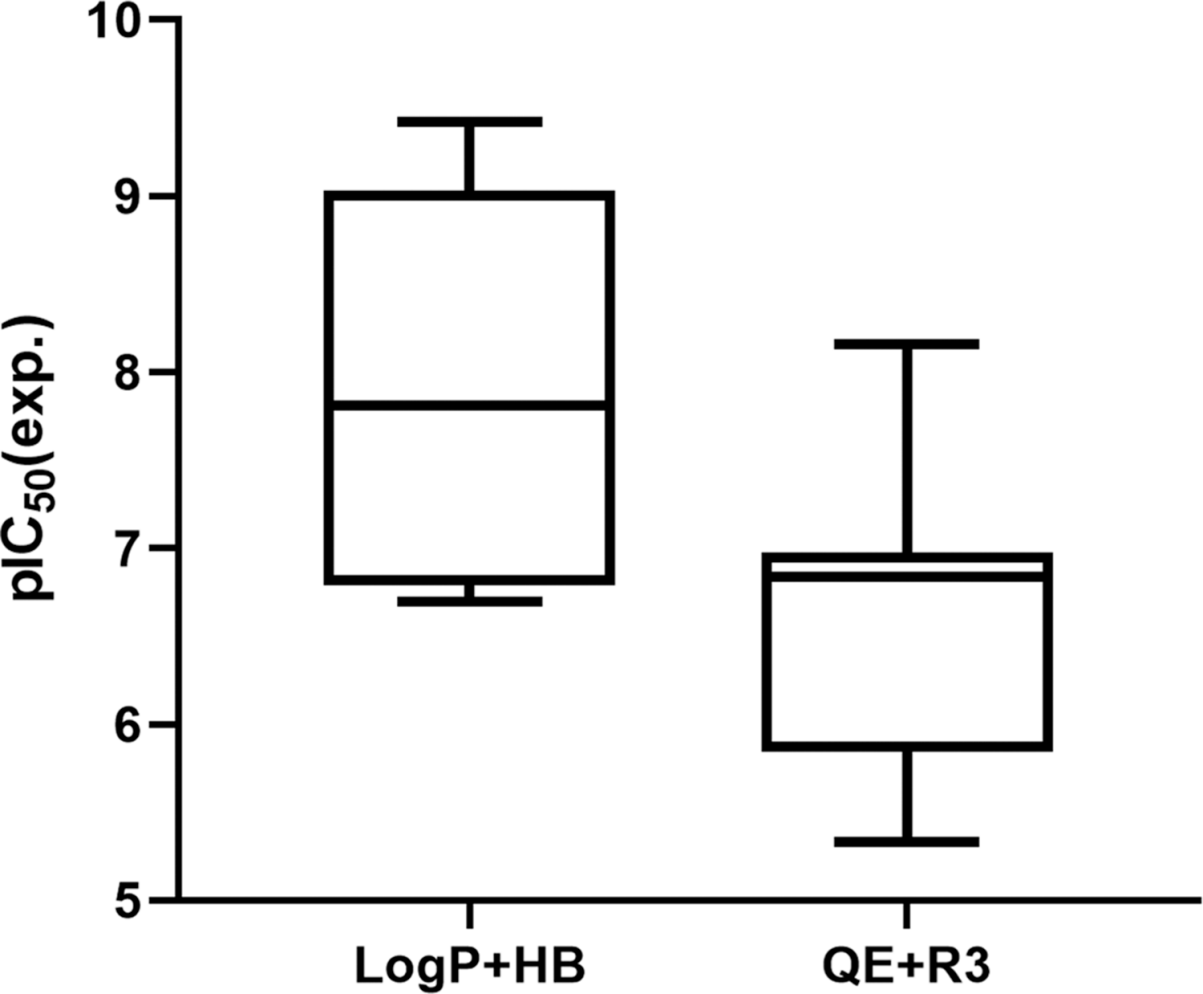
Boxplot of the distribution of pIC_50_ values determined for the groups of compounds selected using the log *P* + HB and QE + R3 (nine for each model) pharmacophores. The horizontal line within the rectangle represents the median of all of the values and the vertical line goes from the maximum to the minimum value.

Figure 9 shows the distribution of experimental activities against the hsEH determined for the nine compounds reranked with the log *P* + HB and QE + R3 pharmacophores. In the former case, the inhibitory potencies range from 6.7 to 9.4, with a mean pIC_50_ value of 7.9 ± 1.0, which is sensibly larger than the averaged pIC_50_ (6.6 ± 0.9) obtained for the compounds selected with the QE + R3 pharmacophore. It is worth noting that the selection of compounds guided by the log *P* + HB pharmacophore leads to five compounds with a potency lower than 20 nM (Figure 9), including two compounds (AK-968/41927527 and Z436130862) with subnanomolar activity. In contrast, the inhibitory potencies determined for the compounds selected by using the QE + R3 model ranges include a single compound with IC_50_ < 20 nM (Z44611499).

Table 3 reports the experimental inhibitory potencies determined for the 18 compounds for the human, rat, and mouse sEH enzymes. The pIC_50_ values exhibit only slight differences between the three enzymes as the differences are generally lower than 0.5 pIC_50_ units, and only in six cases, the difference in the inhibitory potency in msEH/rsEH relative to hsEH is close to 1 pIC_50_ unit (Z41644030, Z237696036, Z23294293, Z44611499, and Z339670416 in human and mouse enzymes and Z29532165 in human and rat enzymes). In a first approximation, the preservation of the inhibitory potency between the three enzymes can be attributed to the fact that the differences in the binding pocket imply the replacement of similar residues, such as the replacement of Met339, Ile363, Leu408, Met419, Leu428, and Met469 in hsEH by Val, Met, Phe, Val, Ile, and Val in both msEH and rsEH, respectively (Figure S5).

**Table 3:** Experimental pIC_50_ Values of the Compounds Included in the Prospective Study by Combining the Nine Best Candidates Selected from Both QE + R3 and log *P* + HB Pharmacophores^a^

compound	hsEH	msEH	rsEH
log *P* + HB-Selected Compounds			
Z211852480	6.9	6.2	6.3
**AK-968/41927527**	**9.4**	8.9	9.4
Z211761694	6.7	6.3	6.7
**Z339843288**	**8.9**	8.9	9.0
**Z416144030**	**7.8**	6.7	7.7
**Z436130862**	**9.1**	9.0	9.0
**Z1499294365**	**7.7**	7.1	7.4
Z237696036	6.7	5.3	6.4
**Z353003074**	**8.2**	8.0	8.3
QE + R3-Selected Compounds			
Z29532165	6.3	6.4	7.4
AK-968/15603026	6.9	6.5	7.4
AO-476/43362680	5.4	4.6	4.6
AK-968/40204236	6.8	5.8	6.5
Z23294293	7.0	6.2	7.4
**Z44611499**	**8.2**	6.9	7.7
Z1033382608	6.3	5.5	6.4
Z339670416	7.0	5.8	6.3
Z65159029	5.3	4.7	5.7

a Compounds with an inhibitory potency against hsEH lower than 20 nM are highlighted in bold.

**Figure 10:**
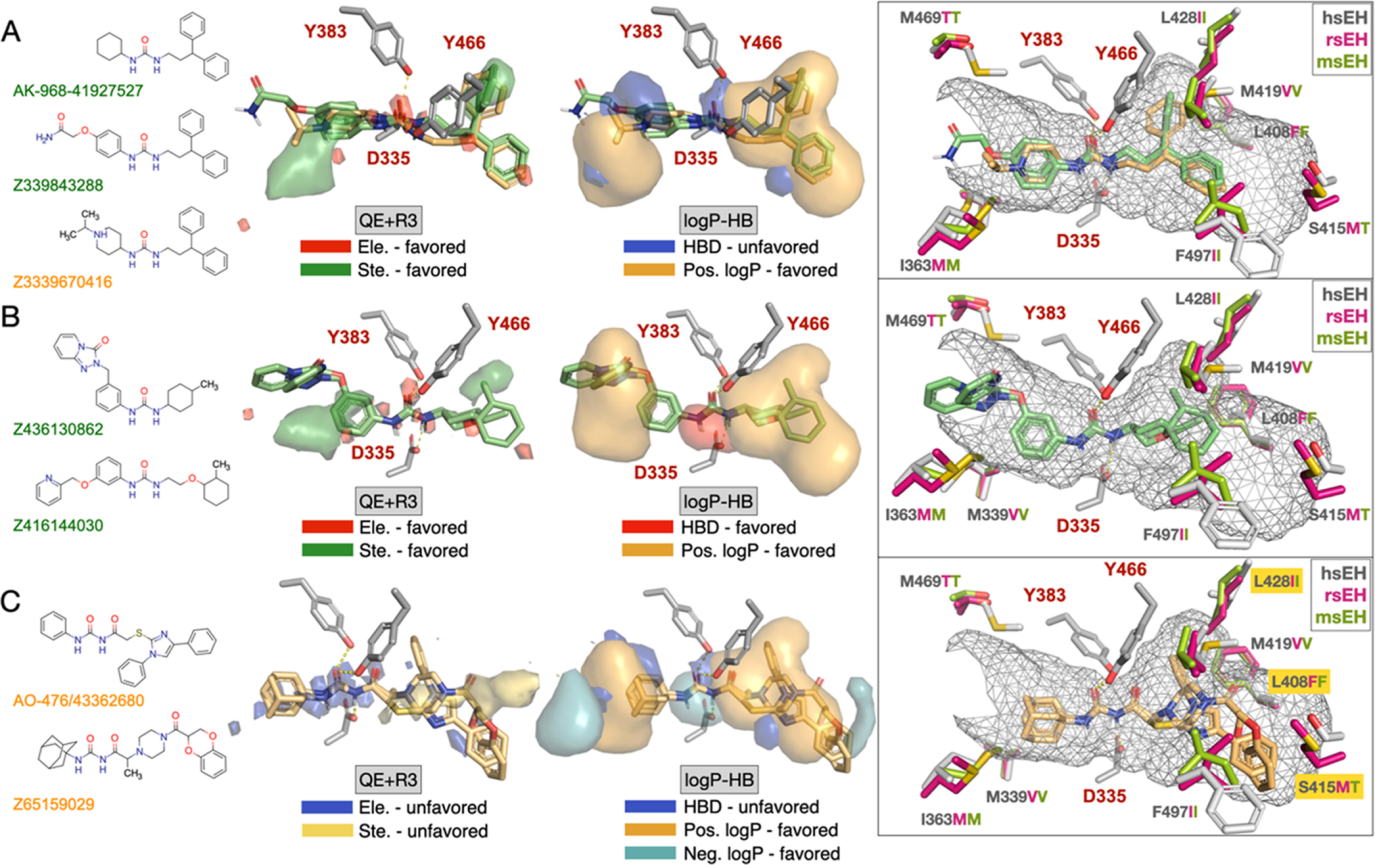
Chemical structures, isocontour maps, and binding modes in the human (gray), rat (pink), and mouse (green) sEH enzymes for (A) 3,3-diphenylpropyl-urea (AK-968-41927527, Z339843288, Z3339670416), (B) three-substituted phenyl-urea (Z436130862, Z416144030), and (C) 2-substituted acetyl-urea (AO-476/43362680, Z65159029) derivatives. Compounds in green/gold were selected by using the log *P* + HB/QE + R3 models, respectively. The PDB ID 3WKE/1CQZ was used as reference for human and mouse enzymes, and the AlphaFold structure with the code AF-P80299-F1-model_v4 was used for the rat sEH.

To calibrate the predictive power of the pharmacophore models, the docking-proposed binding mode in the human, mouse, and rat enzymes was examined for selected compounds (Figure 10).

### 3,3-Diphenylpropyl-Urea Derivatives

AK-968/41927527, Z339843288, and Z3339670416 share a 3,3-diphenylpropyl-urea moiety, the two former being selected according to the log *P* + HB reranking and the latter from the QE + R3 model (Figure 10A). AK-968-41927527 and Z339843288 have experimental pIC_50_ values of 9.4/8.9/9.4 and 8.9/8.9/9.0 for the h/m/rsEH enzymes, respectively (Figure 10A), and the inhibitory potency of Z3339670416 is weaker by at least two orders of magnitude (pIC_50_ values of 7.0/5.8/6.3 for h/m/rsEH enzymes). A close look at the isocontour maps (Figure 10A, middle) revealed that the positively charged nitrogen of this latter compound occupies a region surrounded by hydrophobic residues (Ile363 and Met339 in hsEH, which change to Met and Val in both mouse and rat enzymes) present in the binding site (left pocket in Figure S6). Whereas the electrostatic field in the QE + R3 model does not penalize the presence of a charged atom in that region, the protonated nitrogen is located in an area (blue and yellow orange isocontours in Figure 10A) where the presence of polar atoms would contribute negatively to activity. This would explain, at least in part, the lower experimental activity within the series for compound Z3339670416. Finally, superposition of the three compounds in the binding site of the human, mouse, and rat enzymes (Figure 9A, right) supports the feasibility of the binding mode due to the absence of clashes caused by the mutated residues, thus justifying the similar inhibitory potency.

### Three-Substituted Phenyl-Urea Derivatives

Z436130862 and Z416144030 (Figure 10B; left), which share a phenyl-urea moiety and were selected by the log *P* + HB model, have experimental pIC_50_ values of 9.1/9.0/9.0 and 7.8/6.7/7.7 for the h/m/rsEH enzymes. Both the QE + R3 and log *P* + HB models support the favorable impact on activity promoted by the presence of bulky and hydrophobic substituents (green and yellow orange isocontours) at the two sides of the central urea moiety (Figure 10B; middle). Compared to compound Z436130862, the lower inhibition measured for Z416144030 cannot be explained from the pharmacophore model, as long as there is a large overlap of the two compounds in the binding pocket. Rather, this may likely be attributed to the fact that the experimental activity was determined for the racemic mixture due to the presence of two chiral centers in the (2-methylcyclohexyl)oxy ethyl moiety. On the other hand, superposition of the two compounds in the binding site of the human, mouse, and rat enzymes (Figure 10B, right) revealed the absence of relevant clashes caused by the mutated residues, thus justifying the similar inhibition potencies obtained for each compound.

### Two-Substituted Acetyl-Urea Derivatives

In contrast to the high inhibitory activity of the previous compounds, the enzymatic assays revealed that AO-476/43362680 and Z65159029, which contain an acetyl-urea moiety (Figure 10C; left), have low inhibitory potencies (pIC_50_ < 5.4 for the three enzymes). The steric field for QE + R3 suggests that the compounds partly occupy a region (yellow isocontour) where the presence of bulky groups is disfavored (Figure 10C; middle). This effect is even more pronounced when the hydrophobic field is considered, since the compounds filled a region (orange isocontour) on the right site of the binding site, where the presence of apolar atoms would be favored (note that the acetyl moiety linked to the piperazinyl-methanone moiety fills the orange region shaped by F497, M419, L408, and L428, which are replaced by Ile, Val, Phe, and Ile in the rat and mouse enzymes, respectively).

## Final Remarks

The presence of a pocket on a biological target is a necessary but not sufficient condition for assessing the suitability to bind drug-like small molecules. This makes it convenient to estimate the likelihood of the molecule binding to the enzyme, which at large extent is determined by the desolvation contribution arising from the release of water molecules from the solvent-occluded regions of both the target’s binding site and the ligand upon the formation of the ligand–protein complex, although evaluating and understanding the role of water molecules in the binding site is still challenging.^75−79^ Noteworthy, this assumption is reinforced by the quantitative relationship found by Cheng et al.^16^ (eq 2) between the maximal achievable binding affinity (Δ*G*_MAP_) that can be attained for the binding pocket and the desolvation of the nonpolar solvent surface area (SASA; *A*_nonpolar_^target^) of the pocket weighted by the ratio between the SASA of the ligand (*A*_druglike_^target^) and the total SASA of the target pocket (*A*_total_^target^) for molecules with similar size and net charge
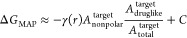
2where γ(*r*) is related to solvent surface tension dependent on the curvature of the pocket surface, and *C* denotes the ligand desolvation component, which can be assumed to be roughly constant for molecules with similar size and net charge.

With this assumption, the physicochemical properties of the hsEH pocket suggest that the use of hydrophobic descriptors may be better suited to explore potential hits targeting the epoxide hydrolase pocket. Indeed, around 80% of the total SASA of the pocket (∼983 Å^2^, as determined with NACCESS calculations^80^) can be ascribed to the presence of nonpolar atoms (see Figure S1). Furthermore, the topological distribution defined by the presence of two wide apolar subpockets separated by the triad of residues that assist the anchoring of the ligand (Tyr383, Tyr466, and Asp335, which is linked to His524) defines a localized character to the polar area in the binding pocket. These features may explain the better performance observed for the identification of potent hsEH inhibitors in the pharmacophore-based reranking of the docked poses obtained for the screened compounds with the hydrophobic descriptors compared with the electrostatic + steric ones. This can be attributed to the precise identification of the isocontours for the hydrophobic field, which clearly shapes regions favorable for the occupancy with polar/apolar groups in the ligand (Figure 4), supplemented by a correction associated to the HB field, which mainly reflects the proper positioning of the polar urea/amide around the anchoring residues in the binding pocket. In contrast, the pharmacophores obtained upon combination of electrostatic and steric fields are more fragmented, making it difficult to interpret the isocontours in terms of the chemical groups that decorate the skeleton of the ligands.

It is also worth to note that the better performance of the Hyphar-derived pharmacophores cannot be anticipated from the results observed for the training dataset, where the QE + R3 model exhibits the best performance as can be observed from the comparison of the LOO regression equation (Figure 3). Rather, the difference between the QE + R3 and log *P* + HB models can be noticed upon analysis of the distinctive behavior found for the two external validation sets, as noted in the larger ability of the latter model to rank the potency of 46 compounds taken from four distinct series of compounds, and the better discrimination between actives and decoys, although the overall performance in early enrichments in this case may be affected by the small size of the decoys in the dataset. Interestingly, the results of this external evaluation point out that the QE + R3 and log *P* + HB models tend to explore distinct chemical spaces, which may open novel opportunities for disclosing unexplored chemical scaffolds and enrich the chemical diversity of drug-like candidates.

The analysis presented in this study supports the suitability of the hydrophobicity field in the virtual screening of chemical libraries and to gain insight into the structure–activity relationships not only for sEH, but also presumably for other targets containing pockets characterized with similar structural and physicochemical properties, as suggested by the results presented in other studies about the impact of hydrophobicity on the binding affinity.^81−83^ In our view, these encouraging results provide a promising basis to explore the range of potential application of the QM-based Hyphar parameters for the retrieval of hits against a wider range of druggable targets.

## Data Availability

PharmQSAR is a proprietary licensed software released by Pharmacelera. It is available in the cloud through a Linux distribution and a REST API. Version 2021.12.R64 (https://pharmacelera.com/pharmqsar/) was used to generate all of the pharmacophore models. Gaussian is licensed software that provides state-of-the-art capabilities for electronic structure modeling released by Gaussian, Inc. In this work, Gaussian16 (https://gaussian.com/) was used to compute the molecular descriptors exploited in this work. Molecular docking was performed with Glide (version 91117), a docking licensed software by Schrodinger, LLC. All of the datasets used in this paper are available in our public GitHub repository at https://github.com/Pharmacelera/Screening_sEH_inhibitors.git.

## Supporting Information Available

Lists of compounds used for the pharmacophore model generation (Table S1), validation sets 1 and 2 (Tables S2 and S3), statistical parameters for subsets of training compounds (Tables S4 and S5), selected virtual hits from Enamine and Specs (Table S6), surface representation of the binding pocket associated to the epoxide hydrolase activity of the hsEH (Figure S1), comparison of the experimental pIC_50_ values versus the fitted ones obtained for the four pharmacophore models (Figure S2), relative distribution of pIC_50_ values for training and test validation sets (Figure S3), and superposition of the structural models for the human (in gray), rat (in pink), and mouse (in green) sEH enzymes (Figure S4) (PDF)
Aligned 3D coordinates for compounds of the training (Table 1_training_set.mol2; 76 compounds), validation set 1 (Table 2_VS1.mol2; 46 compounds), validation set 2 (Table 3_VS2_actives.mol2 and Table 3_VS2_decoys.mol2; 57 + 130 compounds), experimentally tested virtual hits (VHs.mol2) (**MOL2**), and *in silico* predicted activities for all of the previously cited sets (Pharmacophore_models.xlsx) according to the four pharmacophore models (XLSX) are available at https://github.com/Pharmacelera/Screening_sEH_inhibitors.

## Notes

B.D.H. authored patents in this area through the University of California, Davis, and cofounded EicOsis Human Health currently in human trials with a related enzyme inhibitor.

## Abbreviations

B3LYP: Becke 3-parameter Lee–Yang–Parr
hsEH: human soluble epoxide hydrolase
HTVS: high-throughput virtual screening
IEF-PCM/MST: integral equation formalism-polarizable continuum model/Miertus–Scrocco–Tomasi
LOO: leave-one-out
PLS: partial least squares
SAR: structure–activity relationship
XP: eXtra Precision
