# Supplementary material for: Screening and Biological Evaluation of Soluble Epoxide Hydrolase Inhibitors: Assessing the Role of Hydrophobicity in the Pharmacophore-Guided Search of Novel Hits
Source: J Chem Inf Model. 2023 May 4;63(10):3209–25. doi: 10.1021/acs.jcim.3c00301 (PMC10207366; doi:10.1021/acs.jcim.3c00301)
Supplement: Supplementary file 1 — ci3c00301_si_001.pdf [file ci3c00301_si_001.pdf]

SUPPORTING INFORMATION

Screening and biological evaluation of soluble epoxide hydrolase (sEH) inhibitors: Assessing the role of hydrophobicity in the pharmacophore-guided search of novel hits

Javier Vázquez, [1,2,‡,*] *Tiziana Ginex,*[1,‡,*] *Albert Herrero,* [2] *Christophe Morisseau,*[3] *Bruce D. Hammock,*[3] *F. Javier Luque*[4,*]

[1] Departament de Nutrició, Ciències de l'Alimentació i Gastronomia, Facultat de Farmàcia i Ciències de l'Alimentació, Institut de Biomedicina (IBUB), Prat de la Riba 171, 08921 Santa Coloma de Gramenet, Spain

[2] Pharmacelera, Parc Científic de Barcelona (PCB), Baldiri Reixac 4-8, 08028 Barcelona, Spain

[3] Department of Entomology and Nematology, and Comprehensive Cancer Center, University of California, Davis, One Shields Avenue, Davis, CA 95616 USA

[4] Departament de Nutrició, Ciències de l'Alimentació i Gastronomia, Facultat de Farmàcia i Ciències de l'Alimentació, Institut de Biomecidina (IBUB) and Institut de Química Teòrica i Computacional (IQTCUB), Prat de la Riba 171, 08921 Santa Coloma de Gramenet, Spain

Content:

Table S1. List of crystallographic and non-crystallographic hsEH inhibitors used in this work.

Table S2. List of sEH inhibitors included in the validation set 1 (**VS1**).

Table S3. List of non-crystallographic sEH inhibitors included in the validation set 2 (**VS2).**

Table S4. Statistical parameters of the pharmacophore models obtained for the compounds in the training set without data for racemic compounds (TR_mol12, TR_mol13, TR_mol44).

Table S5. Statistical parameters of the pharmacophore models obtained for the compounds in the training set without data characterized by the inhibition constant (TR_mol10, TR_mol11, TR_mol65-68).

Table S6. List of 18 virtual hits selected from Enamine and SPECS and selected physicochemical parameters calculated with ADMETlab 2.0.

Figure S1. Surface representation of the binding pocket associated to the epoxide hydrolase activity of the hsEH.

Figure S2. Comparison of the experimental pIC_{50} values versus the fitted ones obtained for the four pharmacophore models.

Figure S3. Distribution of pIC_{50} values for compounds in (**A**) the training dataset and in the validation subsets VS1 (**B**; in yellow) and VS2 (**B**; in green).

Figure S4. Superposition of the structural models for the human (in grey), rat (in pink) and mouse (in green) sEH enzymes.

Table S1. List of crystallographic and non-crystallographic hsEH inhibitors used in this work.

Number	PDB entry	Ligand name	Code (this work)	Resolution	pIC_{50}	Net Charge	Reference publication
1	3OTQ	MZL	TR_mol1	3.00	7.4	0	10.1016/j.bmcl.2010.09.095
2	3PDC	ZYI	TR_mol2	2.60	7.5	0	10.1021/jm101382t
3	3WK4	S0A	TR_mol3	2.11	5.1	0	10.1016/j.bmc.2014.03.001
4	3WK5	S0C	TR_mol4	2.77	4.8	0	10.1016/j.bmc.2014.03.001
5	3WKE	AUB	TR_mol5	2.75	8.9	-1	10.1016/j.bmc.2014.03.001
6	4C4Y	7WI	TR_mol6	2.41	5.4	0	10.1002/anie.201500671
7	4C4Z	W9L	TR_mol7	2.06	4.0	0	10.1002/anie.201500671
8	4HAI	I23	TR_mol8	2.55	8.1	0	10.1016/j.bmcl.2012.11.084
9	4JNC	1LF	TR_mol9	1.96	8.5	0	10.1016/j.bmcl.2013.04.019
10 [b]	4OCZ	2RU	TR_mol10	2.94	9.2	0	10.1021/jm500694p
11 [b]	4OD0	2RV	TR_mol11	2.92	9.0	0	10.1021/jm500694p
12 [a]	4X6X	S74	TR_mol12	1.80	8.6	-1	10.1016/j.bmcl.2015.02.076
13 [a,c]	4X6Y	S94	TR_mol13	2.10	7.7	0	10.1016/j.bmcl.2015.02.076
14	5AI5	BSU	TR_mol14	2.28	7.0	0	10.1016/j.drudis.2015.04.005
15	5AKE	6N0	TR_mol15	2.26	7.8	0	10.1016/j.drudis.2015.04.005
16	5AKL	6N8	TR_mol16	2.00	7.1	0	10.1016/j.drudis.2015.04.005
17	5ALG	R4N	TR_mol17	2.40	7.5	0	10.1016/j.drudis.2015.04.005
18 [c]	5ALH	4UA	TR_mol18	1.90	7.1	0	10.1016/j.drudis.2015.04.005
19 [c]	5ALI	Q3B	TR_mol19	1.85	7.1	0	10.1016/j.drudis.2015.04.005
20	1ZD4	S127	TR_mol20	-	3.3	0	10.1021/acs.jcim.5b00592
21	1ZD4	S130	TR_mol21	-	3.3	0	10.1021/acs.jcim.5b00592
22	1ZD4	S148	TR_mol22	-	3.3	0	10.1021/acs.jcim.5b00592
23	1ZD3	NC4	TR_mol23	2.30	3.3	-1	10.1110/ps.051720206
24	5ALP	QYD	TR_mol24	2.06	7.1	0	10.1016/j.drudis.2015.04.005
25 [d]	5ALU	HD2	TR_mol25	1.87	7.7	0	10.1016/j.drudis.2015.04.005
26	5ALZ	XQ9	TR_mol26	2.30	8.2	0	10.1016/j.drudis.2015.04.005
27	5AM1	I5T	TR_mol27	2.15	8.5	0	10.1016/j.drudis.2015.04.005
28	6AUM	BXV	TR_mol28	2.95	9.1	-1	10.1016/j.bmcl.2018.01.003
29	6FR2	E3N	TR_mol29	2.26	7.9	0	10.1016/j.bioorg.2018.07.014
30	6HGV	G3Q	TR_mol30	2.00	5.6	1	10.1021/acsmedchemlett.9b00075
31	6HGX	G3T	TR_mol31	2.16	7.1	1	10.1021/acsmedchemlett.9b00075
32	6YL4	OWW	TR_mol32	2.00	8.2	0	10.1021/acs.jmedchem.0c00561
33	1ZD4	NC6	TR_mol33	2.70	3.6	-1	10.1110/ps.051720206
34	3ANS	7	TR_mol34	-	7.2	0	10.1021/jm101273e
35	3ANS	8	TR_mol35	-	7.4	0	10.1021/jm101273e
36	3ANS	9	TR_mol36	-	7.5	0	10.1021/jm101273e
37	3ANS	10	TR_mol37	-	8.6	0	10.1021/jm101273e
38	3I28	4	TR_mol38	-	6.2	0	10.1021/jm9005302
39	3KOO	12	TR_mol39	-	5.8	0	10.1016/j.bmcl.2009.11.091

40	3KOO	14	TR_mol40	-	5.5	0	10.1016/j.bmcl.2009.11.091
41	3KOO	19	TR_mol41	-	6.0	-1	10.1016/j.bmcl.2009.11.091
42	3KOO	24	TR_mol42	-	6.7	0	10.1016/j.bmcl.2009.11.091
43	3KOO	25	TR_mol43	-	6.6	0	10.1016/j.bmcl.2009.11.091
44 [a]	1ZD5	NC7	TR_mol44	2.60	4.9	-1	10.1110/ps.051720206
45	3PDC	1a	TR_mol45	-	7.0	0	10.1021/jm101382t
46	3PDC	1d	TR_mol46	-	7.1	0	10.1021/jm101382t
47	3PDC	1e	TR_mol47	-	6.6	0	10.1021/jm101382t
48	3PDC	1f	TR_mol48	-	6.7	0	10.1021/jm101382t
49	3PDC	1h	TR_mol49	-	8.5	0	10.1021/jm101382t
50	3PDC	1k	TR_mol50	-	7.0	0	10.1021/jm101382t
51	3PDC	1l	TR_mol51	-	6.7	0	10.1021/jm101382t
52	4HAI	7-6'	TR_mol52	-	8.8	0	10.1016/j.bmcl.2012.11.084
53	4HAI	7-12'	TR_mol53	-	7.7	0	10.1016/j.bmcl.2012.11.084
54	4HAI	7-13'	TR_mol54	-	7.7	0	10.1016/j.bmcl.2012.11.084
55	3ANS	S38	TR_mol55	1.98	6.3	0	10.1021/jm101273e
56	4HAI	7-14'	TR_mol56	-	6.6	0	10.1016/j.bmcl.2012.11.084
57	4HAI	7-15'	TR_mol57	-	7.5	0	10.1016/j.bmcl.2012.11.084
58	4HAI	7-16'	TR_mol58	-	6.2	0	10.1016/j.bmcl.2012.11.084
59	4HAI	7-19'	TR_mol59	-	7.3	0	10.1016/j.bmcl.2012.11.084
60	4HAI	7-22'	TR_mol60	-	7.0	0	10.1016/j.bmcl.2012.11.084
61	4HAI	7-23'	TR_mol61	-	8.3	0	10.1016/j.bmcl.2012.11.084
62	4HAI	7-32'	TR_mol62	-	7.6	0	10.1016/j.bmcl.2012.11.084
63	4HAI	7-39'	TR_mol63	-	6.5	0	10.1016/j.bmcl.2012.11.084
64	3ANT	S82	TR_mol64	2.40	8.1	0	10.1021/jm101273e
65 [b]	4OD0	12	TR_mol65	-	8.6	0	10.1021/jm500694p
66 [b]	4OD0	14	TR_mol66	-	9.4	0	10.1021/jm500694p
67 [b]	4OD0	15	TR_mol67	-	8.4	0	10.1021/jm500694p
68 [b]	4OD0	17	TR_mol68	-	9.6	0	10.1021/jm500694p
69	4X6X	12	TR_mol69	-	7.9	-1	10.1016/j.bmcl.2015.02.076
70	4X6X	13	TR_mol70	-	7.1	-1	10.1016/j.bmcl.2015.02.076
71	4X6X	17	TR_mol71	-	7.8	0	10.1016/j.bmcl.2015.02.076
72	4X6X	19	TR_mol72	-	9.2	0	10.1016/j.bmcl.2015.02.076
73	3PDC	1i	TR_mol73	-	8.4	0	10.1021/jm101382t
74	3I1Y	33N	TR_mol74	2.47	8.2	0	10.1021/jm9005302
75	3I28	34N	TR_mol75	1.95	8.2	0	10.1021/jm9005302
76	3KOO	24D	TR_mol76	2.79	7.8	0	10.1016/j.bmcl.2009.11.091

[a] Activity determined for the racemate.
[b] Inhibitory potency given as pK_i.
[c] Compounds containing chemical moieties with poorly defined densities in the X-ray crystallographic structure.
[d] Only the innermost structure of the ligands found in the X-ray structure, which interacts with the catalytic triad, was retained.

Table S2. List of sEH inhibitors included in the validation set 1 (**VS1**).

Number	Ligand in the reference publication *	Code (this work)	pIC_{50}	Net Charge	Reference publication
1	15	VS1_mol1	6.0	1	10.1021/acs.jmedchem.0c00310
2	18	VS1_mol2	6.3	0	10.1021/acs.jmedchem.0c00310
3	19	VS1_mol3	6.3	0	10.1021/acs.jmedchem.0c00310
4	20	VS1_mol4	6.8	0	10.1021/acs.jmedchem.0c00310
5	21	VS1_mol5	7.3	0	10.1021/acs.jmedchem.0c00310
6	22	VS1_mol6	6.7	0	10.1021/acs.jmedchem.0c00310
7	23	VS1_mol7	6.7	0	10.1021/acs.jmedchem.0c00310
8	24	VS1_mol8	6.5	0	10.1021/acs.jmedchem.0c00310
9	25	VS1_mol9	7.1	0	10.1021/acs.jmedchem.0c00310
10	26	VS1_mol10	6.6	0	10.1021/acs.jmedchem.0c00310
11	14	VS1_mol11	8.1	-1	10.1021/acs.jmedchem.0c00310
12	11d	VS1_mol12	8.2	0	10.1021/jm2001376
13	11f	VS1_mol13	7.1	0	10.1021/jm2001376
14	11g	VS1_mol14	7.1	0	10.1021/jm2001376
15	15	VS1_mol15	7.6	0	10.1021/jm2001376
16	21b	VS1_mol16	8.4	0	10.1021/jm2001376
17	21a	VS1_mol17	8.5	0	10.1021/jm2001376
18	21c	VS1_mol18	8.0	0	10.1021/jm2001376
19	21f	VS1_mol19	9.3	0	10.1021/jm2001376
20	21j	VS1_mol20	9.3	0	10.1021/jm2001376
21	10	VS1_mol21	9.4	0	10.1016/j.bmc.2019.115078
22	11	VS1_mol22	9.4	0	10.1016/j.bmc.2019.115078
23	13	VS1_mol23	8.5	0	10.1016/j.bmc.2019.115078
24	14	VS1_mol24	8.5	0	10.1016/j.bmc.2019.115078
25	15	VS1_mol25	8.1	0	10.1016/j.bmc.2019.115078
26	19	VS1_mol26	8.1	-1	10.1016/j.bmc.2019.115078
27	21	VS1_mol27	9.3	-1	10.1016/j.bmc.2019.115078
28	24	VS1_mol28	8.2	0	10.1016/j.bmc.2019.115078
29	25	VS1_mol29	7.7	0	10.1016/j.bmc.2019.115078
30	26	VS1_mol30	8.5	0	10.1016/j.bmc.2019.115078
31	28	VS1_mol31	8.1	0	10.1016/j.bmc.2019.115078
32	7	VS1_mol32	7.7	0	10.1021/acs.jmedchem.0c00310
33	27	VS1_mol33	6.1	0	10.1021/acs.jmedchem.0c00310
34	28	VS1_mol34	6.0	0	10.1021/acs.jmedchem.0c00310
35	29	VS1_mol35	5.8	0	10.1021/acs.jmedchem.0c00310
36	30	VS1_mol36	7.3	0	10.1021/acs.jmedchem.0c00310
37	31	VS1_mol37	7.7	0	10.1021/acs.jmedchem.0c00310
38	12	VS1_mol38	7.5	0	10.1021/acs.jmedchem.0c00310
39	7g	VS1_mol39	6.5	0	10.1021/acs.jmedchem.1c01331

40	8b	VS1_mol40	8.3	0	10.1021/acs.jmedchem.1c01331
41	8c	VS1_mol41	8.5	0	10.1021/acs.jmedchem.1c01331
42	8d	VS1_mol42	8.1	0	10.1021/acs.jmedchem.1c01331
43	8e	VS1_mol43	8.3	0	10.1021/acs.jmedchem.1c01331
44	8f	VS1_mol44	8.2	0	10.1021/acs.jmedchem.1c01331
45	9a	VS1_mol45	6.8	0	10.1021/acs.jmedchem.1c01331
46	9b	VS1_mol46	7.9	0	10.1021/acs.jmedchem.1c01331

Table S3. List of non-crystallographic sEH inhibitors included in the validation set 2 (**VS2**).[a]

Number	Code (original paper)	Code (this work)	pIC_{50}	Net charge	Stereochemistry	Reference publication
			**Actives**			
1	S11	VS2_mol1	9.1	1	-	10.1016/j.bmcl.2009.05.102
2	S12	VS2_mol2R	>9.0	0	R	10.1016/j.bmcl.2009.07.138
3	S12	VS2_mol2S	>9.0	0	S	10.1016/j.bmcl.2009.07.138
4	S13	VS2_mol3	8.6	0	-	10.1016/j.bmcl.2009.08.074
5	S14	VS2_mol4	8.4	0	-	10.1016/j.bmcl.2009.11.091
6	S15	VS2_mol5	8.4	0	-	10.1016/j.bmcl.2009.08.074
7	S16	VS2_mol6	9.0	1	-	10.1016/j.bmcl.2010.03.074
8	S17	VS2_mol7	9.0	0	-	10.1016/j.bmcl.2010.03.074
9	S18	VS2_mol8	9.0	0	-	10.1016/j.bmcl.2010.03.074
10	S19	VS2_mol9	9.0	0	-	10.1016/j.bmcl.2010.04.078
11	S1	VS2_mol10R	9.2	0	R	10.1016/j.bmc.2006.09.057
12	S1	VS2_mol10S	9.2	0	S	10.1016/j.bmc.2006.09.057
13	S20	VS2_mol11	8.5	0	-	10.1016/j.bmcl.2010.04.078
14	S21	VS2_mol12	8.3	0	-	10.1016/j.bmcl.2010.09.095
15	S22	VS2_mol13	9.3	0	-	10.1021/jm2001376
16	S23	VS2_mol13	7.0	0	-	10.1021/jm030514j
17	S24	VS2_mol14	9.4	0	-	10.1021/jm100691c
18	S25	VS2_mol15	9.4	0	-	10.1021/jm100691c
19	S26	VS2_mol16	8.9	0	-	10.1016/j.ejps.2010.03.018; 10.1021/jm070705c
20	S27	VS2_mol17	9.0	0	-	10.1021/jm070705c
21	S28	VS2_mol18	9.2	-1	-	10.1021/jm070270t; 10.1016/j.ejps.2010.03.018
22	S2	VS2_mol19R	8.0	0	R	10.1016/j.bmcl.2009.01.013
23	S2	VS2_mol19S	8.0	0	S	10.1016/j.bmcl.2009.01.013
24	S30	VS2_mol20	7.8	-1	-	10.1021/jm900634w
25	S32	VS2_mol21	9.0	0	-	10.1021/jm101087u
26	S33	VS2_mol22	8.8	0	-	10.1016/j.bmcl.2011.10.074
27	S34	VS2_mol23	7.9	0	-	10.1158/1535-7163.MCT-09-0119
28	S35	VS2_mol24	7.2	0	-	10.1158/1535-7163.MCT-09-0119
29	S36	VS2_mol25	8.2	0	-	10.1016/j.bmc.2011.07.034
30	S37	VS2_mol26	7.0	0	-	10.1158/1535-7163.MCT-09-0119
31	S38	VS2_mol27	9.4	0	-	10.1016/j.bmc.2011.07.034
32	S39	VS2_mol28	8.8	0	-	10.1016/j.ejps.2010.03.018
33	S3	VS2_mol29	8.7	0	-	10.1016/j.bmc.2006.09.057
34	S40	VS2_mol30	9.0	0	-	10.1016/j.bmc.2011.07.034
35	S41	VS2_mol31	9.3	0	-	10.1016/j.bmc.2011.07.034
36	S42	VS2_mol32	8.7	1	-	10.1016/j.ejps.2010.03.018
37	S43	VS2_mol33	9.4	0	-	10.1016/j.bmc.2011.07.034

38	S44	VS2_mol34	8.5	0	-	10.1021/jm101382t
39	S45	VS2_mol35	8.4	0	-	10.1021/jm101382t
40	S46	VS2_mol36	8.7	0	-	10.1021/jm101431v
41	S47	VS2_mol37	8.6	0	-	10.1016/j.ejps.2010.03.018
42	S49	VS2_mol38	9.4	0	-	10.1016/j.bmc.2011.07.034
43	S4	VS2_mol39	9.0	0	-	10.1016/j.bmcl.2009.01.069; 10.1016/j.ejps.2010.03.018
44	S50	VS2_mol40	8.9	0	-	10.1016/j.ejps.2010.03.018
45	S51	VS2_mol41	8.8	0	-	10.1016/j.ejps.2010.03.018
46	S52	VS2_mol42	9.1	0	-	10.1016/j.ejps.2010.03.018
47	S5	VS2_mol43	9.4	0	-	10.1016/j.bmcl.2006.08.078; 10.1016/j.ejps.2010.03.018
48	S6	VS2_mol44	9.1	1	-	10.1016/j.bmcl.2009.01.013; 10.1016/j.bmcl.2009.05.102
49	S7	VS2_mol45R	>9.0	0	R	10.1016/j.bmcl.2009.07.138
50	S7	VS2_mol45S	>9.0	0	S	10.1016/j.bmcl.2009.07.138
51	S9	VS2_mol46	9.0	0	-	10.1021/jm900725r
52	13	VS2_mol47	9.1	0	-	10.1016/j.bmcl.2010.12.042
53	14	VS2_mol48	7.2	0	-	10.1158/1535-7163.MCT-09-0119
54	15	VS2_mol49	9.4	0	-	10.1016/j.bmc.2011.07.034
55	16	VS2_mol50	7.0	0	-	10.1021/jm0500929
56	17	VS2_mol51	7.0	0	-	10.1021/jm030514j
57	18	VS2_mol52	7.0	-1	-	10.1158/1535-7163.MCT-09-0119
**Decoys [b]**						
58	S100	VS2_mol53		0	-	10.1021/acs.jcim.5b00592
59	S103	VS2_mol54		1	-	10.1021/acs.jcim.5b00592
60	S104	VS2_mol55		1	-	10.1021/acs.jcim.5b00592
61	S106	VS2_mol56		0	-	10.1021/acs.jcim.5b00592
62	S110	VS2_mol57		-1	-	10.1021/acs.jcim.5b00592
63	S113	VS2_mol58		0	-	10.1021/acs.jcim.5b00592
64	S114	VS2_mol59		0	-	10.1021/acs.jcim.5b00592
65	S115	VS2_mol60		0	-	10.1021/acs.jcim.5b00592
66	S116	VS2_mol61		0	-	10.1021/acs.jcim.5b00592
67	S117	VS2_mol62		0	-	10.1021/acs.jcim.5b00592
68	S118	VS2_mol63		0	-	10.1021/acs.jcim.5b00592
69	S119	VS2_mol64		0	-	10.1021/acs.jcim.5b00592
70	S120	VS2_mol65		0	-	10.1021/acs.jcim.5b00592
71	S122	VS2_mol66		0	-	10.1021/acs.jcim.5b00592
72	S123	VS2_mol67		0	-	10.1021/acs.jcim.5b00592
73	S124	VS2_mol68		0	-	10.1021/acs.jcim.5b00592
74	S125	VS2_mol69		0	-	10.1021/acs.jcim.5b00592
75	S126	VS2_mol70		0	-	10.1021/acs.jcim.5b00592
76	S128	VS2_mol71		0	-	10.1021/acs.jcim.5b00592

77	S131	VS2_mol72		0	-	10.1021/acs.jcim.5b00592
78	S132	VS2_mol73		0	-	10.1021/acs.jcim.5b00592
79	S133	VS2_mol74		0	-	10.1021/acs.jcim.5b00592
80	S134	VS2_mol75		0	-	10.1021/acs.jcim.5b00592
81	S135	VS2_mol76		0	-	10.1021/acs.jcim.5b00592
82	S136	VS2_mol77		0	-	10.1021/acs.jcim.5b00592
83	S138	VS2_mol78		0	-	10.1021/acs.jcim.5b00592
84	S139	VS2_mol79		0	-	10.1021/acs.jcim.5b00592
85	S140	VS2_mol80		0	-	10.1021/acs.jcim.5b00592
86	S142	VS2_mol81		0	-	10.1021/acs.jcim.5b00592
87	S144	VS2_mol82		0	-	10.1021/acs.jcim.5b00592
88	S145	VS2_mol83		0	-	10.1021/acs.jcim.5b00592
89	S146	VS2_mol84		0	-	10.1021/acs.jcim.5b00592
90	S150	VS2_mol85		0	-	10.1021/acs.jcim.5b00592
91	S151	VS2_mol86		0	-	10.1021/acs.jcim.5b00592
92	S152	VS2_mol87		0	-	10.1021/acs.jcim.5b00592
93	S153	VS2_mol88		1	-	10.1021/acs.jcim.5b00592
94	S154	VS2_mol89		0	-	10.1021/acs.jcim.5b00592
95	S155	VS2_mol90		0	-	10.1021/acs.jcim.5b00592
96	S156	VS2_mol91		-1	-	10.1021/acs.jcim.5b00592
97	S157	VS2_mol92		0	-	10.1021/acs.jcim.5b00592
98	S158	VS2_mol93		0	-	10.1021/acs.jcim.5b00592
99	S159	VS2_mol94		-1	-	10.1021/acs.jcim.5b00592
100	S160	VS2_mol95		-1	-	10.1021/acs.jcim.5b00592
101	S161	VS2_mol96		-1	-	10.1021/acs.jcim.5b00592
102	S162	VS2_mol97		0	-	10.1021/acs.jcim.5b00592
103	S163	VS2_mol98		-1	-	10.1021/acs.jcim.5b00592
104	S164	VS2_mol99		0	-	10.1021/acs.jcim.5b00592
105	S165	VS2_mol100		-1	-	10.1021/acs.jcim.5b00592
106	S166	VS2_mol101		0	-	10.1021/acs.jcim.5b00592
107	S168	VS2_mol102		0	-	10.1021/acs.jcim.5b00592
108	S170	VS2_mol103		0	-	10.1021/acs.jcim.5b00592
109	S171	VS2_mol104		0	-	10.1021/acs.jcim.5b00592
110	S173	VS2_mol105		0	-	10.1021/acs.jcim.5b00592
111	S174	VS2_mol106		0	-	10.1021/acs.jcim.5b00592
112	S175	VS2_mol107		0	-	10.1021/acs.jcim.5b00592
113	S176	VS2_mol108		0	-	10.1021/acs.jcim.5b00592
114	S177	VS2_mol109		0	-	10.1021/acs.jcim.5b00592
115	S178	VS2_mol110		-1	-	10.1021/acs.jcim.5b00592
116	S179	VS2_mol111		0	-	10.1021/acs.jcim.5b00592
117	S180	VS2_mol112		0	-	10.1021/acs.jcim.5b00592
118	S181	VS2_mol113		0	-	10.1021/acs.jcim.5b00592
119	S182	VS2_mol114		0	-	10.1021/acs.jcim.5b00592

120	S183	VS2_mol115		0	-	10.1021/acs.jcim.5b00592
121	S184	VS2_mol116		0	-	10.1021/acs.jcim.5b00592
122	S185	VS2_mol117		0	-	10.1021/acs.jcim.5b00592
123	S186	VS2_mol118		0	-	10.1021/acs.jcim.5b00592
124	S189	VS2_mol119		0	-	10.1021/acs.jcim.5b00592
125	S190	VS2_mol120		0	-	10.1021/acs.jcim.5b00592
126	S191	VS2_mol121		0	-	10.1021/acs.jcim.5b00592
127	S192	VS2_mol122		0	-	10.1021/acs.jcim.5b00592
128	S202	VS2_mol123		0	-	10.1021/acs.jcim.5b00592
129	S205	VS2_mol124		0	-	10.1021/acs.jcim.5b00592
130	S224	VS2_mol125		0	-	10.1021/acs.jcim.5b00592
131	S225	VS2_mol126		0	-	10.1021/acs.jcim.5b00592
132	S226	VS2_mol127		0	-	10.1021/acs.jcim.5b00592
133	S227	VS2_mol128		0	-	10.1021/acs.jcim.5b00592
134	S229	VS2_mol129		0	-	10.1021/acs.jcim.5b00592
135	S230	VS2_mol130		1	-	10.1021/acs.jcim.5b00592
136	S234	VS2_mol131		-1	-	10.1021/acs.jcim.5b00592
137	S236	VS2_mol132		0	-	10.1021/acs.jcim.5b00592
138	S237	VS2_mol133		0	-	10.1021/acs.jcim.5b00592
139	S238	VS2_mol134		0	-	10.1021/acs.jcim.5b00592
140	S239	VS2_mol135		0	-	10.1021/acs.jcim.5b00592
141	S240	VS2_mol136		0	-	10.1021/acs.jcim.5b00592
142	S242	VS2_mol137		0	-	10.1021/acs.jcim.5b00592
143	S243	VS2_mol138		0	-	10.1021/acs.jcim.5b00592
144	S53	VS2_mol139		0	-	10.1021/acs.jcim.5b00592
145	S54	VS2_mol140		-2	-	10.1021/acs.jcim.5b00592
146	S55	VS2_mol141		-1	-	10.1021/acs.jcim.5b00592
147	S56	VS2_mol142		0	-	10.1021/acs.jcim.5b00592
148	S57	VS2_mol143		-2	-	10.1021/acs.jcim.5b00592
149	S58	VS2_mol144		-1	-	10.1021/acs.jcim.5b00592
150	S59	VS2_mol145		-1	-	10.1021/acs.jcim.5b00592
151	S60	VS2_mol146		-1	-	10.1021/acs.jcim.5b00592
152	S61	VS2_mol147		-1	-	10.1021/acs.jcim.5b00592
153	S62	VS2_mol148		-1	-	10.1021/acs.jcim.5b00592
154	S63	VS2_mol149		-1	-	10.1021/acs.jcim.5b00592
155	S64	VS2_mol150		-1	-	10.1021/acs.jcim.5b00592
156	S65	VS2_mol151		-1	-	10.1021/acs.jcim.5b00592
157	S66	VS2_mol152		-1	-	10.1021/acs.jcim.5b00592
158	S67	VS2_mol153		-1	-	10.1021/acs.jcim.5b00592
159	S68	VS2_mol154		-1	-	10.1021/acs.jcim.5b00592
160	S69	VS2_mol155		-2	-	10.1021/acs.jcim.5b00592
161	S70	VS2_mol156		0	-	10.1021/acs.jcim.5b00592
162	S71	VS2_mol157		-1	-	10.1021/acs.jcim.5b00592

163	S72	VS2_mol158		-2	-	10.1021/acs.jcim.5b00592
164	S73	VS2_mol159R		-1	R	10.1021/acs.jcim.5b00592
165	S73	VS2_mol159S		-1	S	10.1021/acs.jcim.5b00592
166	S74	VS2_mol160		-1	-	10.1021/acs.jcim.5b00592
167	S75	VS2_mol161		-1	-	10.1021/acs.jcim.5b00592
168	S76	VS2_mol162R		-2	R	10.1021/acs.jcim.5b00592
169	S76	VS2_mol162S		-2	S	10.1021/acs.jcim.5b00592
170	S77	VS2_mol163		0	-	10.1021/acs.jcim.5b00592
171	S78	VS2_mol164		-2	R	10.1021/acs.jcim.5b00592
172	S79	VS2_mol165		0	-	10.1021/acs.jcim.5b00592
173	S80	VS2_mol166		0	-	10.1021/acs.jcim.5b00592
174	S81	VS2_mol167		-1	-	10.1021/acs.jcim.5b00592
175	S83	VS2_mol168		1	-	10.1021/acs.jcim.5b00592
176	S84	VS2_mol169		0	-	10.1021/acs.jcim.5b00592
177	S86	VS2_mol170		0	-	10.1021/acs.jcim.5b00592
178	S87	VS2_mol171		0	-	10.1021/acs.jcim.5b00592
179	S88	VS2_mol172		0	-	10.1021/acs.jcim.5b00592
180	S89	VS2_mol173		1	-	10.1021/acs.jcim.5b00592
181	S90	VS2_mol174		-1	-	10.1021/acs.jcim.5b00592
182	S91	VS2_mol175		-1	-	10.1021/acs.jcim.5b00592
183	S92	VS2_mol176		0	-	10.1021/acs.jcim.5b00592
184	S96	VS2_mol177		1	-	10.1021/acs.jcim.5b00592
185	S97	VS2_mol178		0	-	10.1021/acs.jcim.5b00592
186	S98	VS2_mol179		0	-	10.1021/acs.jcim.5b00592
187	S99	VS2_mol180		0	-	10.1021/acs.jcim.5b00592

[a] Data taken from ref. 62. Compounds already included in the training set, without the urea/formamide (or structurally similar units) or without specific annotation of the stereochemical species were excluded in this study.

[b] Molecules characterized by intramolecular atomic clashes or unable to give a correct placement of the urea/formamide moiety around the catalytic triad (S102, S129, S137, S141, S143, S147, S149, S199, S201, S203, S204, S206-208, S228) were excluded from the original set.

Table S4. Statistical parameters of the pharmacophore models obtained for the compounds in the training set without data for racemic compounds (TR_mol12, TR_mol13, TR_mol44).

Parameter	QG+LJ	QE+R3	logP	logP+HB
N_c	7	7	3	3
Field (%)	QG: 10<br>R3: 90	QE: 53<br>R3: 47	logP: 100	logP: 79<br>HBD: 14<br>HBA: 7
Regression model y = m x + n				
m	0.51	0.87	0.84	0.88
n	3.69	0.72	1.22	0.90
q^2	0.33	0.67	0.62	0.57
Spress	1.28	0.88	0.92	0.99
Regression model y = c x				
c	1.03	0.98	1.01	1.00
r^2	0.95	0.99	0.98	0.98

Table S5. Statistical parameters of the pharmacophore models obtained for the compounds in the training set without data characterized by the inhibition constant (TR_mol10, TR_mol11, TR_mol65-68).

Parameter	QG+LJ	QE+R3	logP	logP+HB
N_c	3	7	3	3
Field (%)	QG: 9<br>R3: 91	QE: 55<br>R3: 45	logP: 100	logP: 80<br>HBD: 14<br>HBA: 6
Regression model y = m x + n				
m	0.49	0.88	0.78	0.83
n	3.77	0.71	1.62	1.33
q^2	0.24	0.63	0.56	0.50
Spress	1.27	0.88	0.99	1.05
Regression model y = c x				
c	1.04	0.99	1.02	1.01
r^2	0.96	0.98	0.98	0.98

Table S6. List of 18 virtual hits selected from Enamine and SPECS and selected physicochemical parameters calculated with ADMETlab 2.0.[a]

Code (smiles)	LogS	LogD	LogP	MW	Vol	nHA	nHD	TPSA	nRot	nRing	PAINS	Lipinski
Z211852480 (CC(NC(=O)NCc1ccc(N2CCCC2=O)cc1)c1cccc(Cl)c1)	-4.6	3.0	2.9	371	373	5	2	61	7	3	0	Yes
AK-968/41927527 (O=C(NCCC(c1ccccc1)c1ccccc1)NC1CCCCC1)	-4.6	4.2	5.2	336	375	3	2	41	8	3	0	Yes
Z211761694 (O=C(NCc1ccc(N2CCCC2=O)cc1)NCc1cccc(C(F)(F)F)c1)	-4.4	3.1	2.7	391	376	5	2	61	8	3	0	Yes
Z339843288 (NC(=O)COc1ccc(NC(=O)NCCC(c2ccccc2)c2ccccc2)cc1)	-4.5	3.1	3.5	403	428	6	4	93	11	3	0	Yes
Z416144030 (CC1CCCCC1OCCNC(=O)Nc1cccc(OCc2ccccn2)c1)	-3.8	3.5	3.4	383	404	6	2	72	10	3	0	Yes
Z436130862 (CC1CCC(NC(=O)Nc2cccc(Cn3nc4ccccn4c3=O)c2)CC1)	-6.0	4.1	4.3	379	389	7	2	80	6	4	0	Yes
Z1499294365 (CC(CNC(=O)NCCc1c[nH]c2ncccc12)c1ccc(F)cc1F)	-3.4	3.3	2.8	358	355	5	3	70	8	3	0	Yes
Z237696036 (Cc1ccc(C(O)(CCNC(=O)Nc2ccc(Cl)c(Cl)c2)C(F)(F)F)o1)	-5.1	3.7	4.2	410	349	5	3	74	8	2	0	Yes
Z353003074 (O=C(NCc1ccc(-c2nc3ccccc3s2)o1)NC1CCCC(C(F)(F)F)C1)	-5.4	4.2	4.3	423	389	5	2	67	7	4	0	Yes
Z29532165 (O=C(NCCC(=O)N1CCN(S(=O)(=O)c2ccccc2F)CC1)NC12CC3CC(CC(C3)C1)C2)	-4.2	2.95	2.70	492	471	8	2	99	9	6	0	Yes
AK-968/15603026 (Cc1ccc(C(C)C)c(OCc2ccc(C(=O)NNC(=O)NC34CC5CC(CC(C5)C3)C4)o2)c1)	-5.7	5.26	4.98	465	482	7	3	93	10	6	0	Yes
AO-476/43362680 (O=C(CSc1nc(-c2ccccc2)cn1-c1ccccc1)NC(=O)Nc1ccccc1)	-5.8	4.32	4.66	428	435	6	2	76	9	4	0	Yes
AK-968/40204236 (O=C(N/N=C/c1c2ccccc2cc2ccccc12)NC12CC3CC(CC(C3)C1)C2)	-7.8	4.98	6.52	397	425	4	2	53	5	7	0	Yes
Z23294293 (O=C(COC(=O)c1ccc(N2CCCC2=O)cc1)NC(=O)NC12CC3CC(CC(C3)C1)C2)	-5.0	2.9	3.5	439	439	8	2	105	9	6	0	Yes
Z44611499	-5.4	4.4	4.3	419	436	6	3	79	9	6	0	Yes

(O=C(NNC(=O)c1ccc(COc2ccccc2)cc1)NC12CC3CC(CC(C3)C1)C2)												
Z1033382608 (CC(=O)CCc1ccc(OCC(=O)N2CCCC(CNC(=O)Nc3ccc(C)cc3)C2)cc1)	-4.8	3.0	2.8	451	477	7	2	88	12	3	0	Yes
Z339670416 (CC(C)N1CCC(NC(=O)NCCC(c2ccccc2)c2ccccc2)CC1)	-3.6	3.8	4.1	379	421	4	2	44	9	3	0	Yes
Z65159029 (CC(C(=O)NC(=O)NC12CC3CC(CC(C3)C1)C2)N1CCN(C(=O)C2COc3ccccc3O2)CC1)	-4.1	3.0	3.1	496	496	9	2	100	8	7	0	Yes

[a] logS: logarithm of aqueous solubility; logD: logarithm of the *n*-octanol/water distribution coefficient at pH=7.4; logP: logarithm of the *n*-octanol/water distribution coefficient; MW: molecular weight; Vol: van der Waals volume; nHA: number of hydrogen bond acceptors; nHD: number of hydrogen bond donors; TPSA: topological polar surface area; nRot: number of rotatable bonds; nRing: number of rings; PAINS: Pan Assay Interference Compounds; Lipinski rule-of-five.

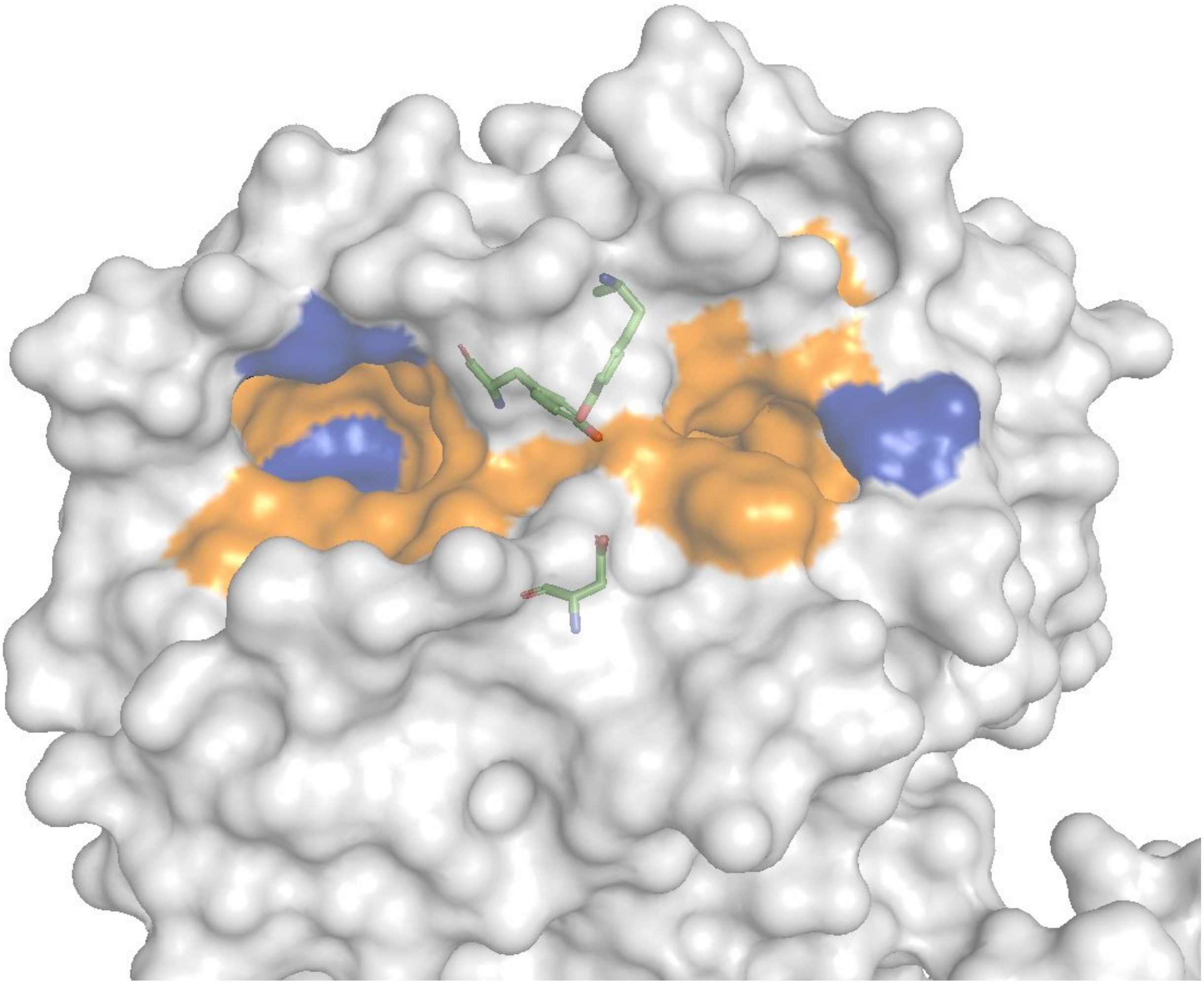

Figure S1. Surface representation of the binding pocket associated to the epoxide hydrolase activity of the hsEH. The residues of the catalytic triad (Tyr383, Tyr466, and Asp335) are shown as green (C atoms) sticks. The surface of the two hydrophobic cavities (Phe267, Pro268, Met310, Trp336, Met339, Pro361, Ile363, Pro364, Pro371, Ile375, Phe381, Phe387, Leu397, Leu408, Leu417, Met419, Leu428, Trp465, Trp469, Phe497, Val498, Leu499, Met503, Trp525) located at both sides of the catalytic triad is shown in orange, and the surface of the polar residues (Thr360, Ser374, Gln384, Ser415, Asn473, His524) is colored in blue.

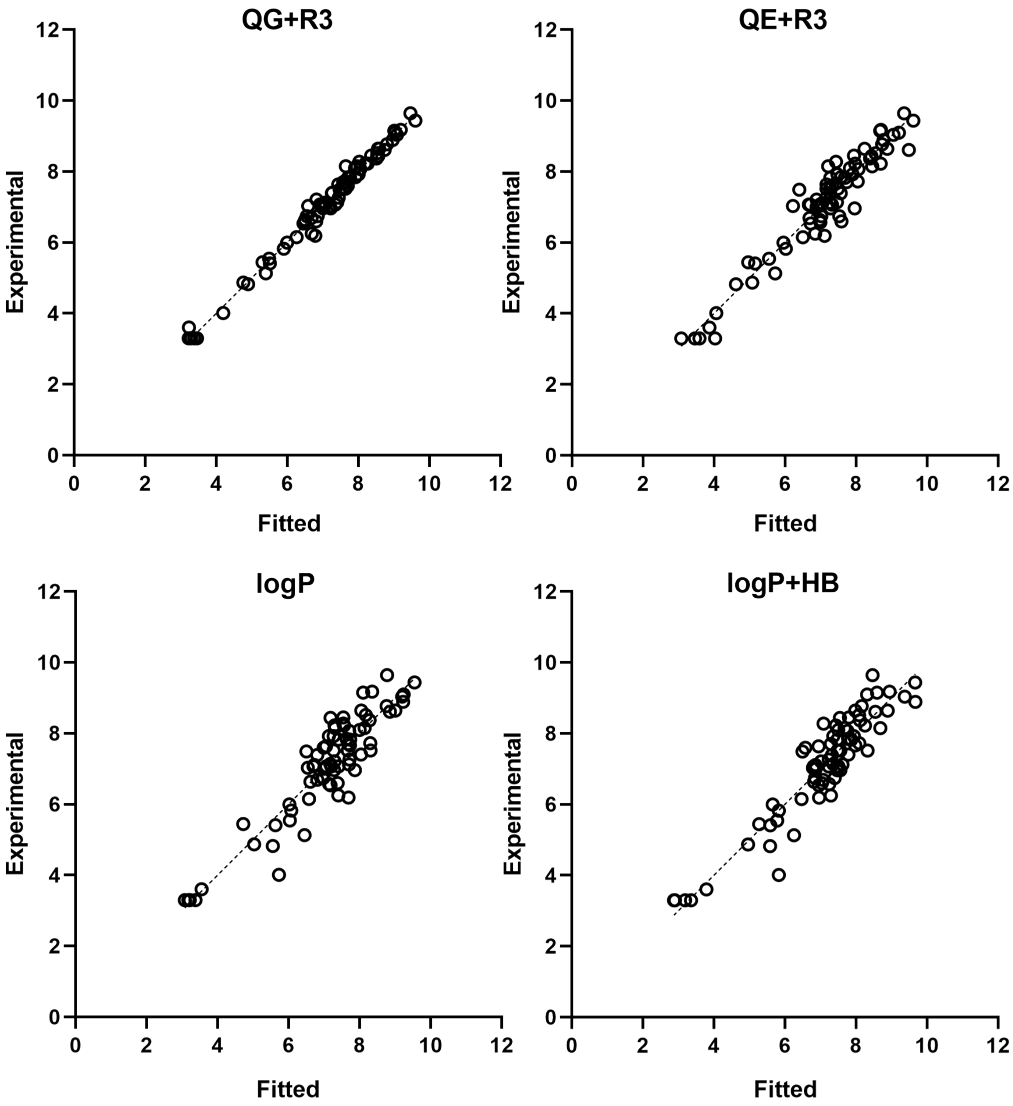

Figure S2. Comparison of the experimental pIC_{50} values versus the fitted ones obtained for the four pharmacophore models.

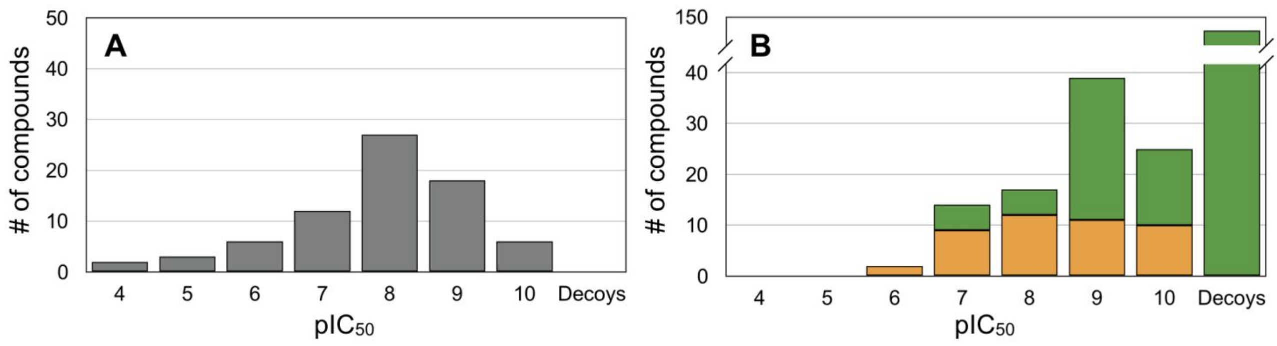

Figure S3. Distribution of pIC_{50} values for compounds in (**A**) the training dataset and (**B**) the validation subsets 1 (yellow) and 2 (green).

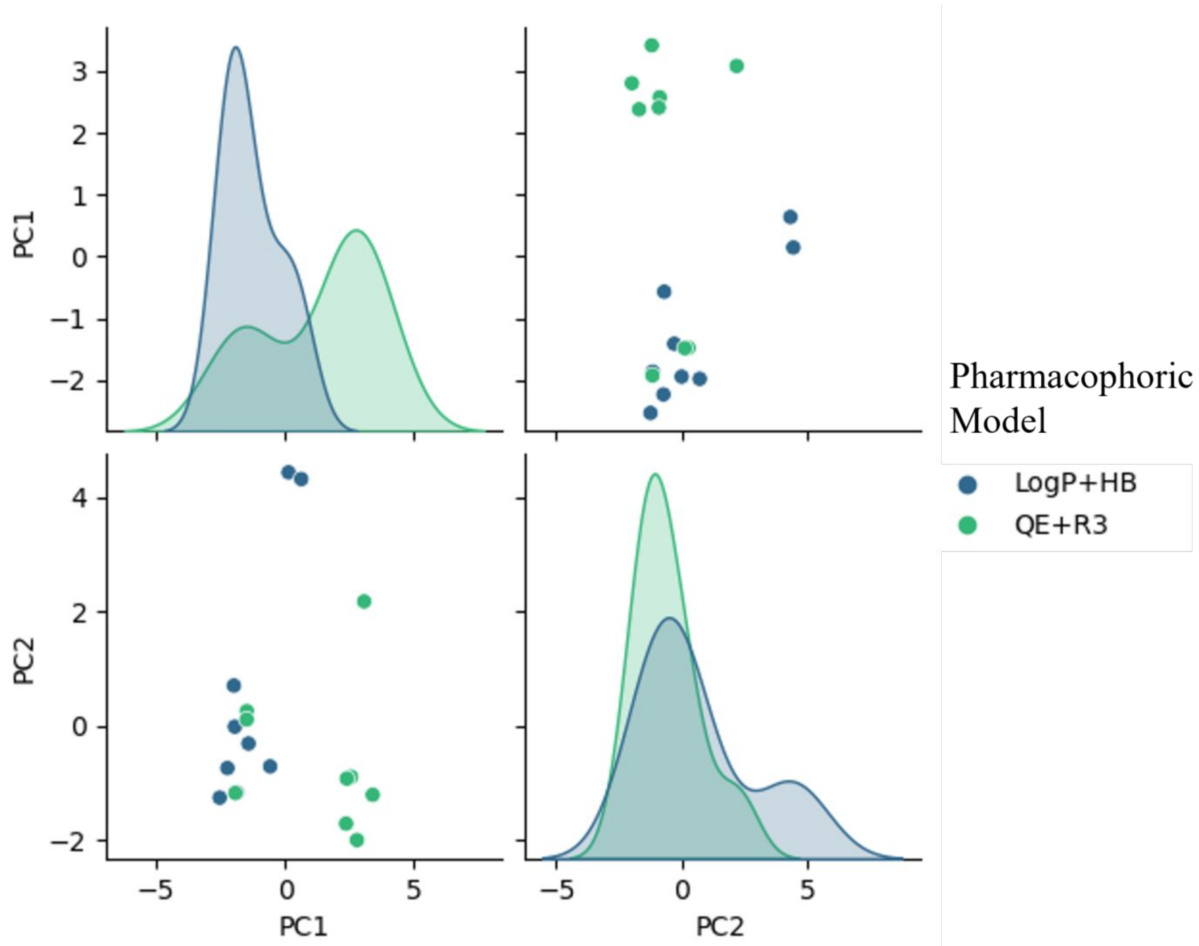

Figure S4. Principal component analysis of the compounds selected in the prospective analysis according to their structural similarity determined by using Morgan Fingerprints radius 2 (1024 bits) as 2D molecular descriptor. Compounds prioritized from the logP+HB and QE+R3 pharmacophores are shown as blue and green dots, respectively.

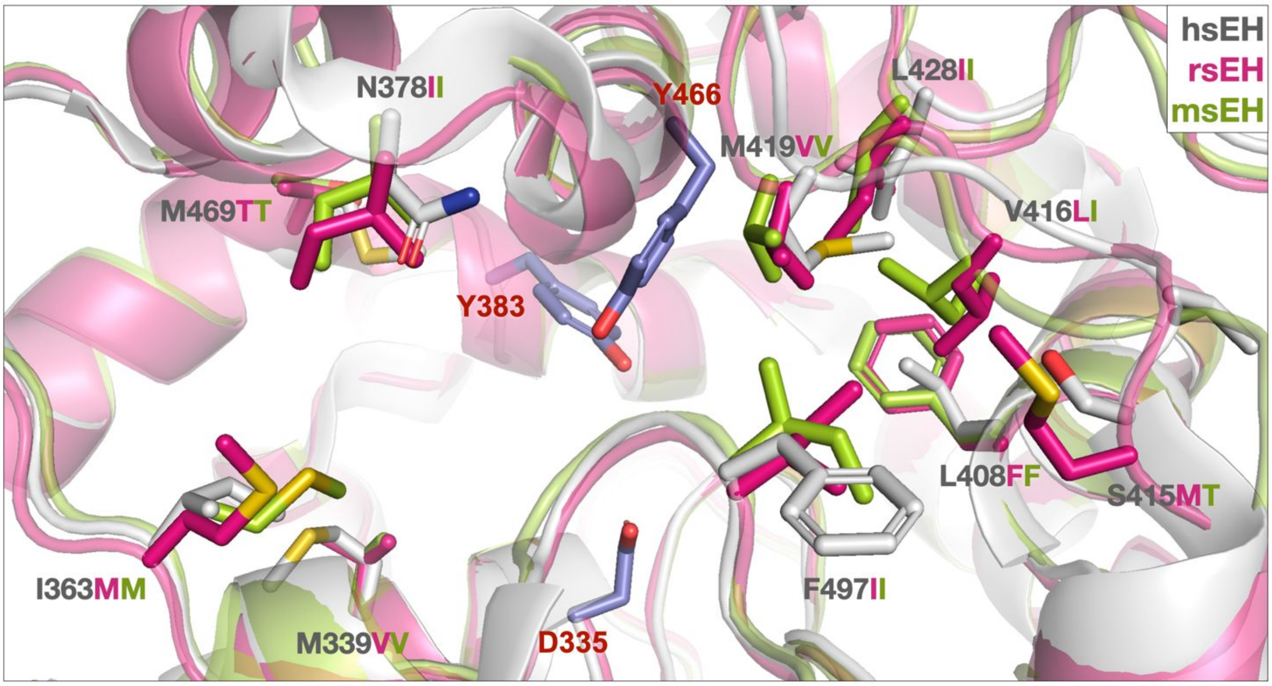

Figure S5. Superposition of the structural models for the human (in grey), rat (in pink) and mouse (in green) sEH enzymes. The PDB ID 3WKE/1CQZ were used for as reference for human and mouse she while the AlphaFold structure with code AF-P80299-F1-model_v4 was used for the rat sEH. Residues of the catalytic triad are highlighted as violet sticks. Amino acid changes among the three systems are highlighted as sticks.
